# N-acetylcysteine (NAC) ameliorates ethanol-induced oxidative stress, neuroinflammation, and cognitive dysfunction in APP/PS1 mouse model

**DOI:** 10.1038/s41398-025-03496-z

**Published:** 2025-10-24

**Authors:** Xiaoyu Pan, Zhengkang Su, Zhengwei Huang, Yue Chen, Xi Li, Xiangtao Zheng

**Affiliations:** 1 Department of Vascular Surgery, The Second Affiliated Hospital of Wenzhou Medical University, Wenzhou, China; 2 The Affiliated Kangning Hospital of Wenzhou Medical University, Zhejiang Clinical Research Center for Mental Disorders, Wenzhou, Zhejiang China

**Keywords:** Molecular neuroscience, Learning and memory

## Abstract

Alzheimer’s disease (AD) is the most prevalent neurodegenerative disorder that predominantly affects the elderly, leading to a progressive decline in cognitive function. Accumulating evidence suggests that many environmental and dietary factors, especially chronic ethanol exposure, aggravate the risk of this disease. However, its precise influence on AD has not yet been clarified. Here, we show that ethanol exposure caused earlier and severer cognitive behavioral impairments, more beta amyloid (Aβ) depositions, microglia activation, decreased total antioxidant capacity (T-AOC). Moreover, inflammatory mediators, such as Nucleotide-binding oligomerization domain-like receptor protein 3 (NLRP3) and Tumor necrosis factor-alpha (TNF-α) increased, while pivotal proteins involved in dendritic and synaptic development, such as Synaptophysin (SYP), postsynaptic density protein 95 (PSD95) and brain-derived neurotrophic factor (BDNF) decreased in APP/PS1 mice. N-acetylcysteine (NAC), a well-known antioxidant, could attenuate cognitive behavioral impairments and neuroinflammatory damage by restoring inflammatory and neurodevelopmental mediators. In general, our study uncovered that chronic ethanol exposure may exacerbate AD progress at the pathological and molecular levels and NAC may act as a potential drug for the treatment of AD patients with chronic ethanol exposure.

## Introduction

Alzheimer’s disease (AD) is a chronic progressive neurodegenerative disease. As the most common form of dementia, AD has a prevalence of 3.48% in elderly individuals in China [1]. The main character of AD is irreversible brain dysfunction, manifested as deterioration of cognitive functions, motor deficits, behavioral disturbances etc. The pathological hallmarks of AD encompass the presence of neurofibrillary tangles (NFTs) and senile plaques (SPs) coupled with neuronal loss and neuroinflammation etc. [2–4]. Despite the profound influence of AD, there is still a lack of curative or fundamentally disease-altering therapies in AD.

Chronic ethanol exposure, a key AD risk factor, triggers neurodegeneration mirroring AD pathology [5, 6]. Over 70% of patients suffered from chronic ethanol exposure which exhibits a certain degree of brain pathology, similar to those who are susceptible to aging and AD [7, 8]. The precise mechanism of how chronic ethanol exposure is modulating AD pathology is yet to be fully uncovered.

Recent study has found that chronic ethanol exposure may alter APP processing and aggravate AD-associated phenotypes [6]. The core of the prevailing theories on the etiology of AD is the amyloid cascade hypothesis. It proposes that the accumulation of β-amyloid (Aβ) peptides in the brain is one of the main factors leading to AD, which further causes early damage to dendritic and synaptic, thus leading to neuronal dysfunction [9]. Beyond its direct impact on neurons, Aβ has also been demonstrated to activate microglia [10], the immune cell of the central nervous system triggering the release of inflammatory mediators [11, 12]. These mediators include the inflammasome complex NOD-like receptor thermal protein domain associated protein 3 (NLRP3), and tumor necrosis factor-alpha (TNF-α), which in turn exacerbate the dendritic and synaptic impairments leading to neuronal death and promote AD progression [13–15]. Clinical study found spatially accumulated activated microglia around Aβ deposits in brain slices from AD patients [16]. Meanwhile, research has shown that Aβ may also induce the generation of reactive oxygen species (ROS) through various pathways, causing lipid peroxidation and neuronal oxidative stress, which causes further neuroinflammatory injury [17–19]. Glutathione (GSH) plays an important role in maintaining the redox state of cells. N-acetylcysteine (NAC), the main contributor to maintain cellular GSH status, can minimize the oxidative effects of ROS by correcting or preventing GSH depletion [20–23].

In this study, we aim to explore whether chronic ethanol exposure leads to oxidative stress-mediated exacerbation of AD pathology, which in turn results in activation of microglia and neuroinflammatory damage, which ultimately leads to cognitive behavioral disorders. And NAC was employed to ameliorate ethanol exposure, which could induce oxidative stress damage and cognitive behavioral impairments in APP/PS1 mouse model.

## Materials and methods

All animal experiments were approved by the Animal Care and Use Committee of Wenzhou Medical University and Ethics Committees of Wenzhou Medical University. All methods were performed in accordance with the guidelines and regulations of the Animal Care and Use Committee of Wenzhou Medical University and Ethics Committees of Wenzhou Medical University. Outcome assessment blinding: Data analysts (e.g., pathological scoring, video tracking) accessed only de-identified codes.

### Mice obtain breeding

The wild-type (WT) C57BL/6 mice were obtained from Beijing Viton Lihua Laboratory Animal Technology Co Ltd, and APP/PS1 double transgenic (2 × Tg) mice were obtained from Hangzhou Ziyuan Laboratory Animal Technology Co Ltd. All animals were housed at Animal Experiment Center of Wenzhou Medical University under the following conditions: 3 mice/ cage (30 × 20 × 15 cm, length × width × height), 12 h dark/light cycle, room temperature (24 ± 1) °C, ambient humidity (50 ± 10) %, with free access to food and water.

### Ethanol administration

Two-month-old mice were used to undergo ethanol administration for 10 weeks. The details of four ethanol exposure paradigms were listed below.

### Drinking in the dark (DID)

The standard DID ethanol drinking paradigm was used in mice to model human binge-like consumption behavior and was described in previous research [24]. Briefly, mice were individually housed in standard laboratory cages. In the first hour of darkness, no drinking water was provided, and one hour later, ethanol was provided for 4 h continuously. The concentration of ethanol solution varied with the modeling time, increasing from 5–20% (mice were offered with 5% ethanol (v/v) for 1 week, then 10% ethanol (v/v) for the next 2 weeks, and 15% ethanol (v/v) for the next 3 weeks, finally animals had access to 20% ethanol (v/v) for last 4 weeks). The control group mice were provided with the same water after 1 h of water withdrawal.

### Two-bottle choice (2BC)

The standard 2BC paradigm was as previous reported [25]. Briefly, mice were housed individually in standard laboratory cages equipped with two bottles, one containing water and the other containing a solution of ethanol. Two drinking tubes were continuously available to each mouse. Mice were exposed to progressively increased concentrations of ethanol as described above. The position of the bottles is switched daily to avoid any potential side preference. The control group mice were provided with two bottles of water.

### Chronic intermittent ethanol (CIE)

Briefly, mice were housed individually in standard laboratory cages. All mice were able to intermittently obtain one bottle of ethanol and one bottle of water with progressively increased concentrations of ethanol and water as described above (24-h period, 4 days/week). The control group mice were provided with water.

### Intraperitoneal injection (IP)

Mice were injected with ethanol (3 mL/kg, 20% v/v solution, intraperitoneal (i.p.)) at intervals every week. Within a week, 20% ethanol was injected for the first two days, followed by an interval of one day, and then ethanol was injected for the next two days. The control group of mice was injected with saline accordingly.

### Behavior experiments

Animals were excluded from statistical analysis if data collection failed due to technical errors (e.g., equipment malfunction, improper sample handling, or missed experimental procedures). All exclusions were documented in the raw dataset, with final group sizes explicitly reported in the Results section. Animals/samples were randomly allocated to experimental groups using a computer-generated random number sequence.

### Locomotor activity (LA) test

The spontaneous locomotor activity was examined to rule out the interference from impaired motor ability on cognitive behavior detection. In short, the mice were placed in a grid of the spontaneous activity tester (30 × 30 × 30 cm) for a total of 15 min. The mice were allowed to adapt to the environment in the first 5 min, and the activities of the mice were recorded for the next 10 min. EthoVision XT10 software was used to record the total locomotion counts. After each experiment, the area was wiped clean with 75% ethanol to avoid interference from odors.

### Novel object recognition (NOR) test

The NOR test was conducted as follows: on day one, mice were habituated to the test box (65 × 45 cm), being allowed to adapt to the environment in 5 min. On day two, mice were placed into the same test box in the presence of two identical objects (6 cm away from the side wall) for 20 min, after an inter-trial interval of 1 h, one familiar object was replaced with a novel object. Mice were placed back within the testing area for 5 min. The exploration time spent on each object (A1, B1) was measured, the total time was recorded as E1. The same procedure was replicated after a 24-h interval. The exploration time spent on each object (A2, B2) was measured, the total time was recorded as E2. After each test, the box was cleaned with 75% ethanol to eliminate odors. Discrimination index (DI) = B–A/E.

### Y-maze test

To examine spatial working memory, spontaneous alternation (SA) in the Y-maze was performed. The main device of the maze consists of three V-shaped opaque arms (40 × 3 × 12 cm) at an angle of 120° to each other, marked as I, II, and III. Mice were placed in the center of the maze and allowed to explore the maze freely for 5 min. The standard for entering each arm was that all four limbs entered. Mice entering three different arms in order (such as I II III, I III II, II III I, II I III, III II I, III I II) was viewed as an alternation. The number and order of mice entering and exiting the three arms were recorded. Spontaneous alternation rate (%) = [(spontaneous alternation number)/ (total number of arm entries–2)] × 100%.

### Morris water maze (MWM) test

The MWM is a widely used behavioral test to assess spatial reference memory in rodent. The MWM test protocol takes 6 days and includes two stages: a training stage, and a testing stage. Before each trial, the water temperature was maintained at 25 ± 0.5 °C, followed by addition of titanium dioxide (0.1 g/L) to achieve standardized opacity. The training stage consists of 5 trials per day for 5 consecutive days, where black visual cues of different shapes are attached to the walls of the pool and the platform is submerged 1 cm beneath the water surface in the target quadrant. Mice were placed into the water from one of the four starting points in a random order and the time taken to find the platform was recorded. If a mouse failed to locate the platform within 1 min, the experimenter gently guided it onto the platform and allowed 10s of spatial orientation. On day 6, probe trials were conducted 1 and 24h post-training. During these trials, the escape platform was removed, and each mouse was introduced into the pool from the quadrant diametrically opposed to the original target location for a single 60s session. The swimming trajectory, average speed, number of platform crossings, target quadrant stay time, target quadrant distance percentage and other test data were recorded.

### Detection of total antioxidant capacity (T-AOC)

The T-AOC was measured using 2,2’-azino-bis 3-ethylbenzthiazoline-6-sulfonic acid (ABTS) method. Briefly, the 20 mg mouse hippocampal tissue was broken and homogenized in 100 μL pre-cooled 1 × PBS solution and centrifuged at 12,000 g for 5 min at 4 °C to obtain the supernatant. The T-AOC of mouse brain hippocampus tissue was detected using the T-AOC Assay Kit with ABTS method (Beyotime Biotechnology, C2006). The ABTS was oxidized to green ABTS^+^ by appropriate oxidant, which will be inhibited in the presence of antioxidants. The T-AOC can be determined by measuring the absorbance of ABTS^+^ at 734 and 405 nm. The kit used Trolox as a reference standard and the total antioxidant capacity of the sample was calculated based on the standard curve. The total antioxidant capacity of the sample was expressed as mmol/g Trolox equivalent antioxidant capacity (TEAC).

### Detection of ATP level

The ATP Assay Kit (Beyotime Biotechnology, S0026) was used to detect the ATP level in mouse hippocampal tissue. Briefly, 20 mg mouse hippocampal tissue was broken and homogenized in 200 μL ATP tissue lysate and centrifuged at 12,000 g for 5 min at 4 °C to obtain the supernatant. Add 100 μL of ATP detection working solution into the detection well at room temperature for 3–5 min to consume all the original ATP and reduce the background. Then, add 20 μL of the sample and diluted standards into the detection well and measure the relative light unit (RLU) using a SpectraMax 190 full wavelength spectrometer (Molecular Devices Corporation), and ATP level then was calculated based on the standard curve.

### Western blot (WB)

The protein of brain tissue was extracted using lysis buffer (RIPA: PMSF = 100:1) on ice. The lysates were collected and centrifuged at 12,000 g for 20 min to obtain the supernatant. Total proteins were quantified using a BCA protein assay kit (Thermofisher). Equal amounts of protein were separated by 12% SDS-PAGE and transferred to PVDF membranes. The PVDF membranes were blocked with 5% nonfat milk for 1.5 h and then incubated overnight at 4 °C with the following primary antibodies: anti-TNF-α (Abcam, ab300093), anti-NLRP3 (Abcam, ab254360), anti-BDNF (Abcam, ab108319), anti-Synaptophysin (SYP) (Abcam, ab156302) and anti-PSD95 (Abcam, ab238135). Then, the membranes were washed and incubated with Goat Anti-Rabbit IgG (H + L) HRP (Affinity, S0001) or Goat Anti-Mouse IgG HRP (Affinity, S0002) for 1 h. Chemiluminescent signals were detected and analyzed using a Bio-Rad Gel Doc XR^+^ gel imaging system (Bio-Rad).

### Immunohistochemical (IHC)

The IHC staining for Aβ 40 proteins was performed in the mouse brain using Anti-Amyloid beta 40 antibody (Aβ 40) (Servicebio, GB111197). In brief, mouse brain tissue sections were sliced (5 µm thickness) and sections were dewaxed, hydrated, and washed, and endogenous H_2_O_2_ was quenched with 3% H_2_O_2_ at room temperature in the dark for 25 min. Antigen retrieval was performed in citric acid antigen retrieval solution (pH 6.0) with high temperature. Secondary antibody (Goat Anti-Rabbit IgG (H + L) HRP, 1:200) for primary antibody (rabbit anti-Amyloid beta 40, 1:400) were used. Chromogen development was performed using Metal Enhanced DAB Substrate Kit (Solarbio, DA1016).

### Immunofluorescence (IF)

In brief, sections were dewaxed, hydrated, washed, antigen retrieval, and endogenous H_2_O_2_ was quenched with 3% H_2_O_2._ After blocked, incubated overnight at 4 °C with rabbit anti-Iba1 (1:100) (Wako, 016-26461). Then, the membranes were washed and incubated with FITC-conjugated goat anti-rabbit IgG (1:300). The slides were counterstained with 4′,6-diamidino-2-phenylindole (DAPI) and observed under a fluorescence microscope.

### NAC treatment

NAC was dissolved in normal saline to a concentration of 0.3 mg/mL. Mice received intraperitoneal injections of this solution at a dose of 3 mg/kg body weight. The mice were randomly divided into four groups: WT control group, APP/PS1 group, APP/PS1-ethanol group, and ethanol +NAC group. And ethanol modeling was conducted for a total of 10 weeks. Intraperitoneal injection of NAC began in the eighth week of alcohol modeling, once a day, for 2 weeks.

### Statistical analysis

All data were statistically analyzed using GraphPad Prism 9.5.1 software and expressed as mean ± standard error of the mean (SEM). Shapiro-Wilk test was used for normality test. Two-way analysis of variance (ANOVA) or Multiple unpaired t tests were used for comparison between multiple groups, the Tukey’s multiple comparisons or Holm-Šídák tests were used to correct the *p*-values for each group, respectively. When the data for comparison between multiple groups did not meet the normal distribution, the Kolmogorov-Smirnov test was used. Significance was defined as *p < 0.05, **p < 0.01, ***p < 0.001, ****p < 0.0001. All statistically significant statistics are presented in Supplementary Table 1. For behavioral tests, n = 5-6 mice per group; for biochemical and histological analyses, n = 3 mice per group. At least three independent coverslips for in vitro experiments were used per test group. A p-value of less than 0.05 was considered statistically significant.

## Results

### Chronic ethanol exposure induced earlier and severer cognitive behavioral impairments in APP/PS1 mice

**Fig. 1:**
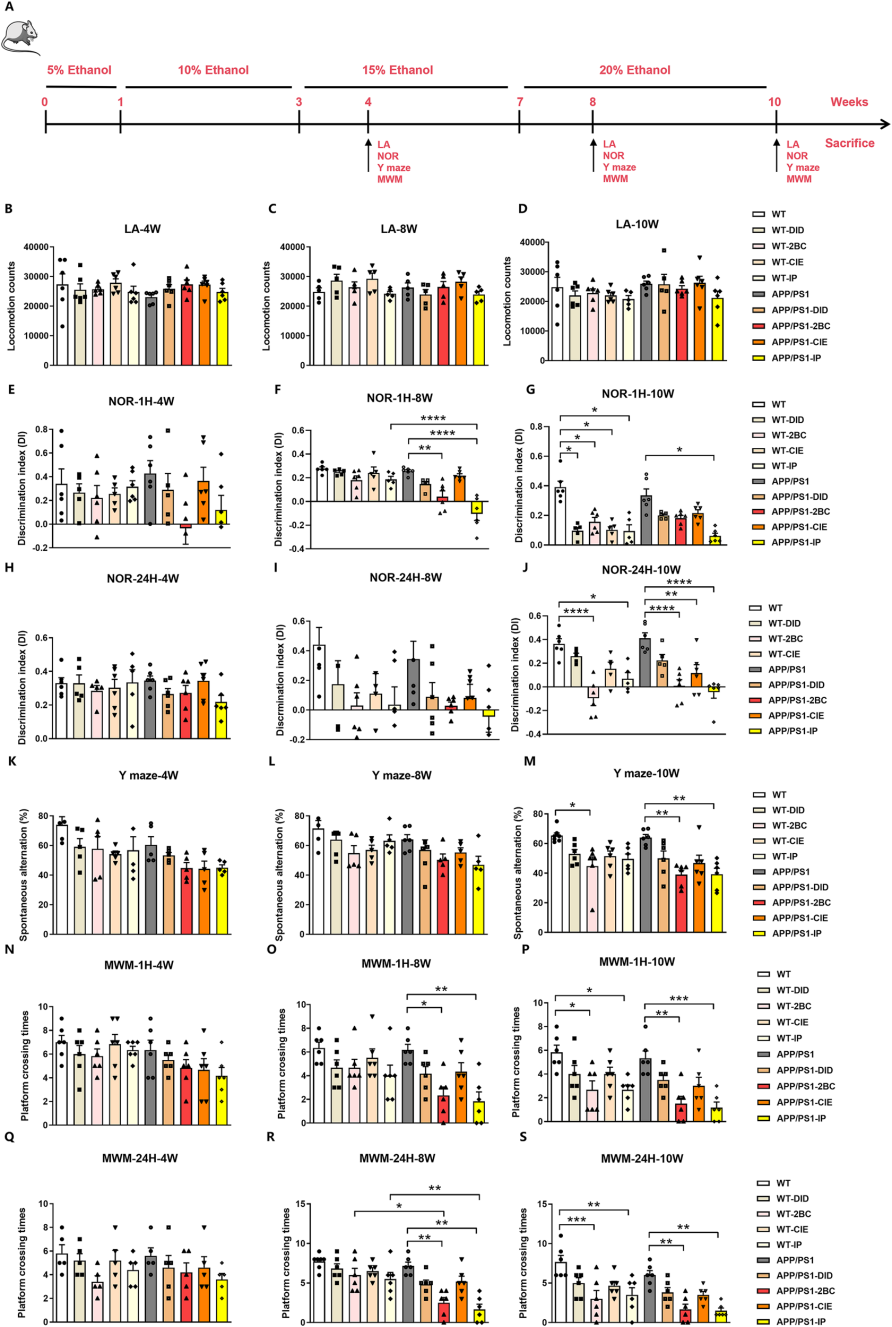
Cognitive behavioral impairments in ethanol-exposed WT and APP/PS1 mice in four paradigms (DID, 2BC, CIE, IP). **A** The schematic illustration of 10-week ethanol exposure process in WT and APP/PS1 mice. **B**–**D** Locomotor activity assessments of WT and APP/PS1 mice in LA test after 4-week, 8-week, and 10-week of ethanol exposure, the spontaneous locomotion activity was detected, no significant difference was found. **E**–**J** The NOR tests were performed to evaluate the short-term (1-h) and long-term (24-h) recognition memory of WT and APP/PS1 mice after 4-week, 8-week, and 10-week of ethanol exposure. The discrimination index (DI) of each group was calculated. **K**–**M** The Y maze was performed to examine spatial working memory of WT and APP/PS1 mice after 4-week, 8-week, and 10-week of ethanol exposure, the spontaneous alternation rate (%) of each group was calculated. **N**–**S** The MWM were performed to examine spatial reference memory of WT and APP/PS1 mice after 4-week, 8-week, and 10-week of ethanol exposure. The platform crossing time 1-h (N-P) and 24-h after training (Q-S) were recorded. Drinking in the dark (DID), two bottle choice (2BC), chronic intermittent ethanol (CIE), and intraperitoneal injection (IP). Each group contains five to six mice, *p < 0.05, **p < 0.01, ***p < 0.001, ****p < 0.0001, *n* = 5 or 6.

To investigate the role of chronic ethanol exposure on cognitive behaviors of wild-type (WT) mice and APP/PS1 mice, continuously increasing ethanol exposure was conducted at three timepoints: 4-week, 8-week, 10-week, and four drinking paradigms: drinking in the dark (DID), two bottle choice (2BC), chronic intermittent ethanol (CIE), and intraperitoneal injection (IP) (Fig. 1A). Firstly, we evaluated the locomotor activity (LA) of the mice and found that there was no significant change in either ethanol exposed WT or APP/PS1 mice (Fig. 1B–D). During the novel object recognition (NOR) tests, gradually deteriorating short-term (1-h) and long-term (24-h) cognitive impairments occurred with the increasing time of ethanol exposure, while earlier and severer recognition memory loss were found in APP/PS1 mice, compared to WT mice (Fig. 1E–J). Likewise, results of Y maze tests demonstrated earlier and more significant impaired spatial working memory in APP/PS1 mice (Fig. 1K–M). Moreover, we detected the alteration of spatial reference memory in Morris water maze (MWM) tests. The reduction in platform crossing times for APP/PS1 occurred earlier and more pronounced at 1 and 24h after training during the test stage (Fig. 1N–S and Supplementary Fig. 1). In general, our result is that chronic ethanol exposure may cause earlier and severer cognitive behavioral impairments in ethanol-exposed APP/PS1 mice.

### Chronic ethanol exposure resulted in earlier appearance of Aβ plaques in APP/PS1 mice

**Fig. 2:**
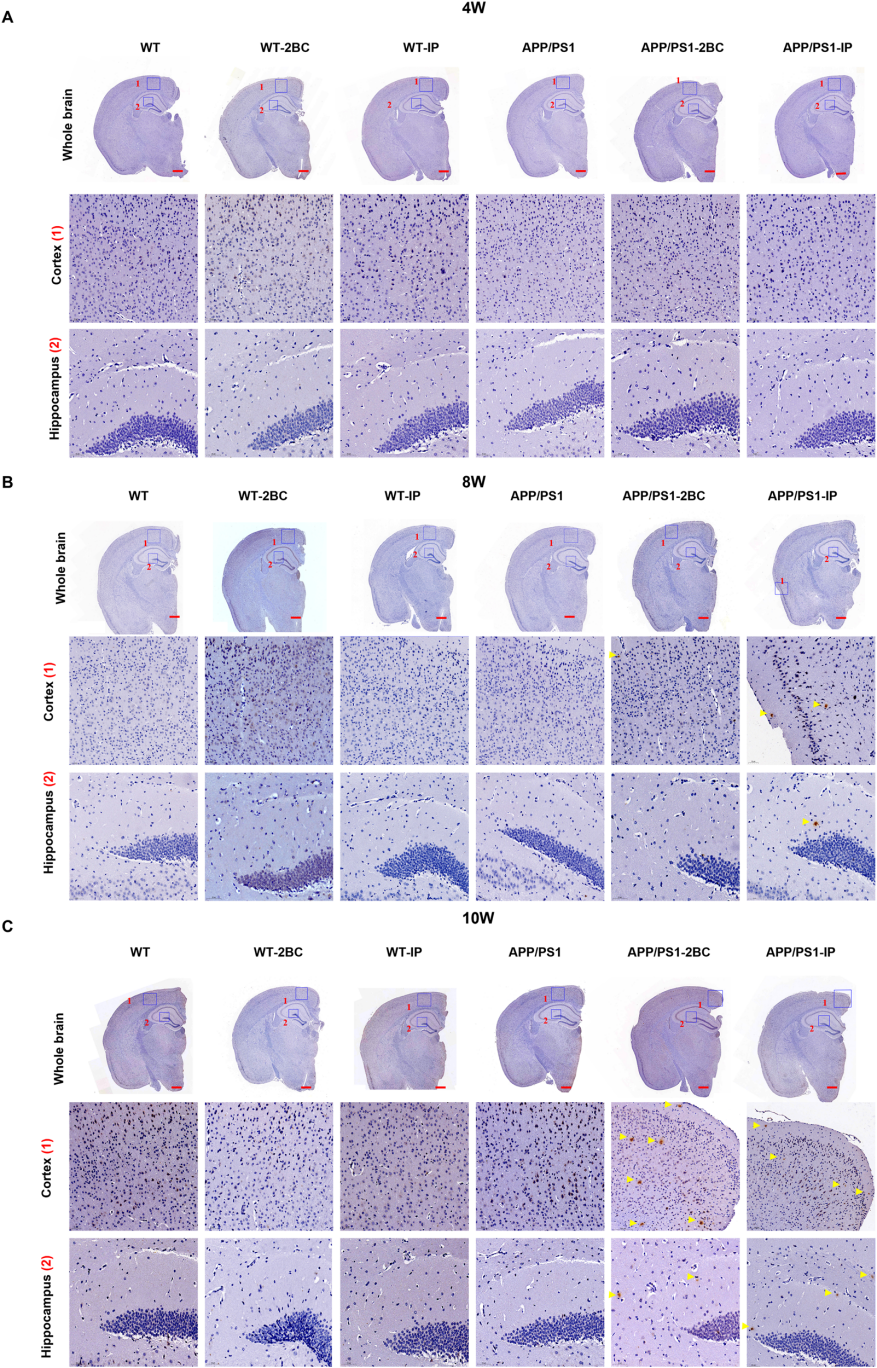
Increased Aβ plaques induced by ethanol exposure in APP/PS1 mice. **A**–**C** Representative images of Aβ plaques immunohistochemistry (IHC) results in the hippocampus and cortex of 4-week, 8-week, and 10-week ethanol-exposed WT and APP/PS1 mice. No Aβ deposition was found in either WT or APP/PS1 mice after 4-week ethanol exposure, while the Aβ depositions appeared in the cortex and hippocampus of 8-week and 10-week ethanol-exposed APP/PS1 mice. Yellow triangle indicated Aβ plaque, scale bar = 500 μm, each group contained three mice.

Previous results have shown more pronounced cognitive behavioral impairments using the 2BC and IP paradigms. The deposition of Aβ in the cortex and hippocampus was investigated in mice subjected to chronic ethanol exposure using 2BC and IP paradigms. Immunohistochemical staining of Aβ showed that no visible Aβ plaque was found after 4-week ethanol exposure in both cortex and hippocampus of WT and APP/PS1 mice (Fig. 2A), while the Aβ plaques appeared in both cortex and hippocampus of APP/PS1 mice after 8-week ethanol exposure (Fig. 2B). Continually exacerbated Aβ deposition was observed in APP/PS1 mice after 10-week ethanol exposure (Fig. 2C), and no Aβ deposition was observed in WT mice. Taken together, chronic ethanol exposure may exacerbate the generation of Aβ deposition and results in earlier appearance of Aβ plaques in APP/PS1 mice.

### Enhanced microglia activation and compromised antioxidant capacity in the hippocampus of ethanol-exposed APP/PS1 mice

**Fig. 3:**
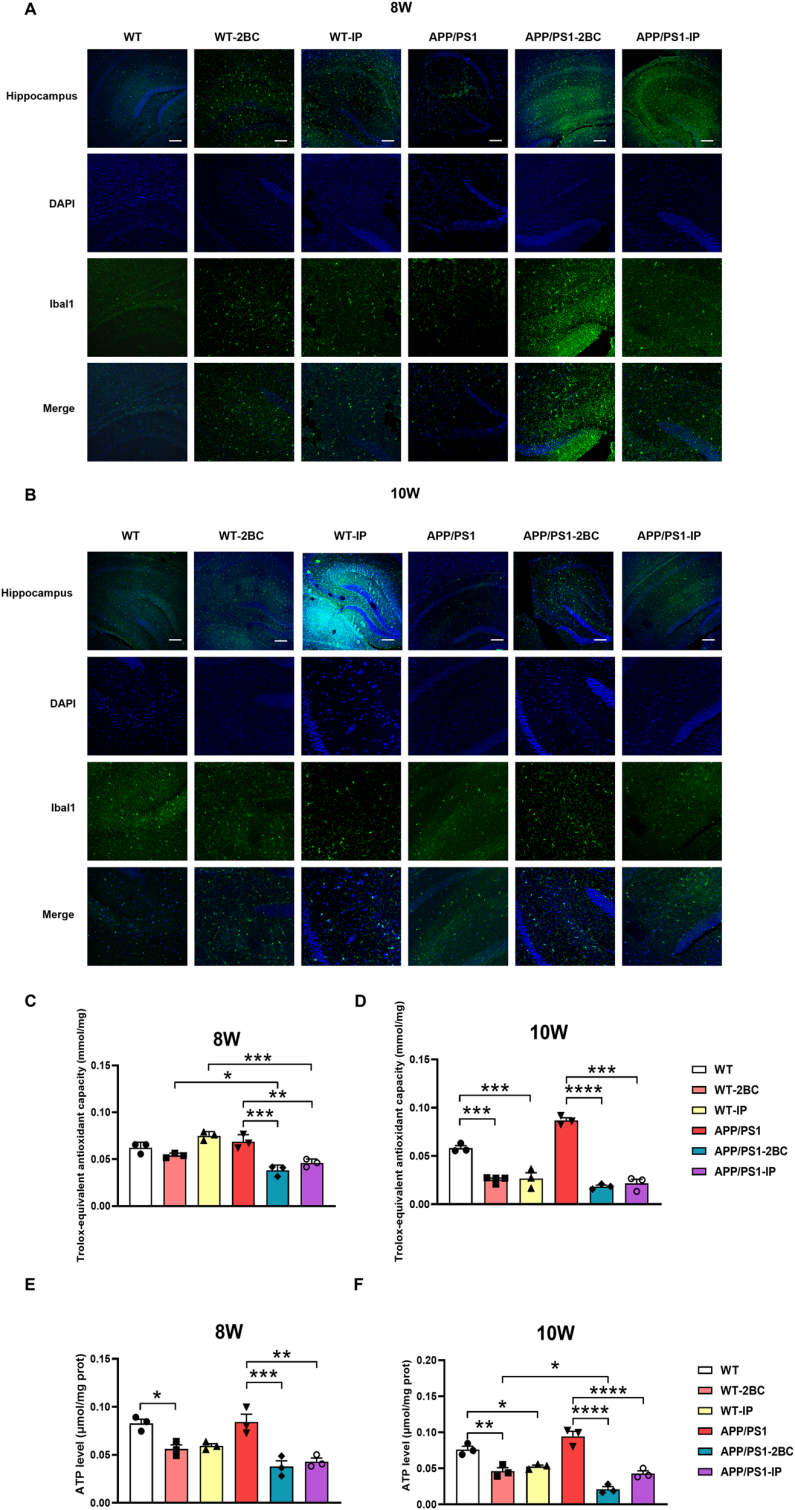
Detection of microglia activation and antioxidant capacity in ethanol exposed WT and APP/PS1 mice. **A,** **B** Representative images of Iba1 immunofluorescence (IF) results in the 8-week and 10-week ethanol-exposed WT and APP/PS1 mice. Iba1 was used as a biomarker of microglia activation. Scale bar = 100 μm. Samples of hippocampus of 8-week and 10-week ethanol-exposed WT and APP/PS1 mice were collected, and the antioxidant capacity (T-AOC) was determined using 2,2’-azino-bis 3-ethylbenzthiazoline-6 -sulfonic acid (ABTS) method. The T-AOC was calculated and presented as trolox-equivalent antioxidant capacity (**C**), (**D**), and downregulated trolox-equivalent antioxidant capacities were found in both 10-week ethanol-exposed WT mice and APP/PS1 mice. **E,** **F** The ATP levels of the hippocampus of 8-week and 10-week ethanol-exposed WT mice and APP/PS1 mice were detected using ATP assay. Reduced ATP levels were found in 8-week and 10-week ethanol-exposed WT mice and APP/PS1 mice. Each group contained three mice. *p < 0.05, **p < 0.01, ***p < 0.001, ****p < 0.0001.

Studies have proved that Aβ depositions may affect activation of microglia [11], we then detected the activation of microglia in mice using Iba1 as a biomarker. The results showed that after 8-week ethanol exposure, increased Iba1^+^ cells were found in the hippocampus of APP/PS1 mice (Fig. 3A). Consistently, a great increased number of Iba1^+^ cells was found in the hippocampus of APP/PS1 mice after 10-week ethanol exposure, and a minor increase was also observed in WT mice (Fig. 3B). The activation of microglia is closely related to oxidative stress and neuroinflammation. We then investigated the total antioxidant capacity (T-AOC) of ethanol-exposed mice. An earlier and greater reduction of T-AOC was observed in the hippocampus of ethanol-exposed APP/PS1 (Fig. 3C, D). A reduction in brain ATP level can impair mitochondrial function, exacerbate oxidative stress and consequently lead to cognitive behavioral deficits [26]. We then investigated the ATP level in the hippocampus of ethanol-exposed mice. And a significantly reduced ATP level was found in both 8-week and 10-week ethanol-exposed WT mice and APP/PS1 mice (Fig. 3E, F). Meanwhile, a greater reduction of ATP level was observed in APP/PS1 mice. Taken together, ethanol exposure is likely to intensify oxidative stress, neuroinflammation and exacerbate compromised antioxidant capacity in APP/PS1 mice compared to WT mice.

### Chronic ethanol exposure caused exacerbated neuroinflammatory impairments in APP/PS1 mice

**Fig. 4:**
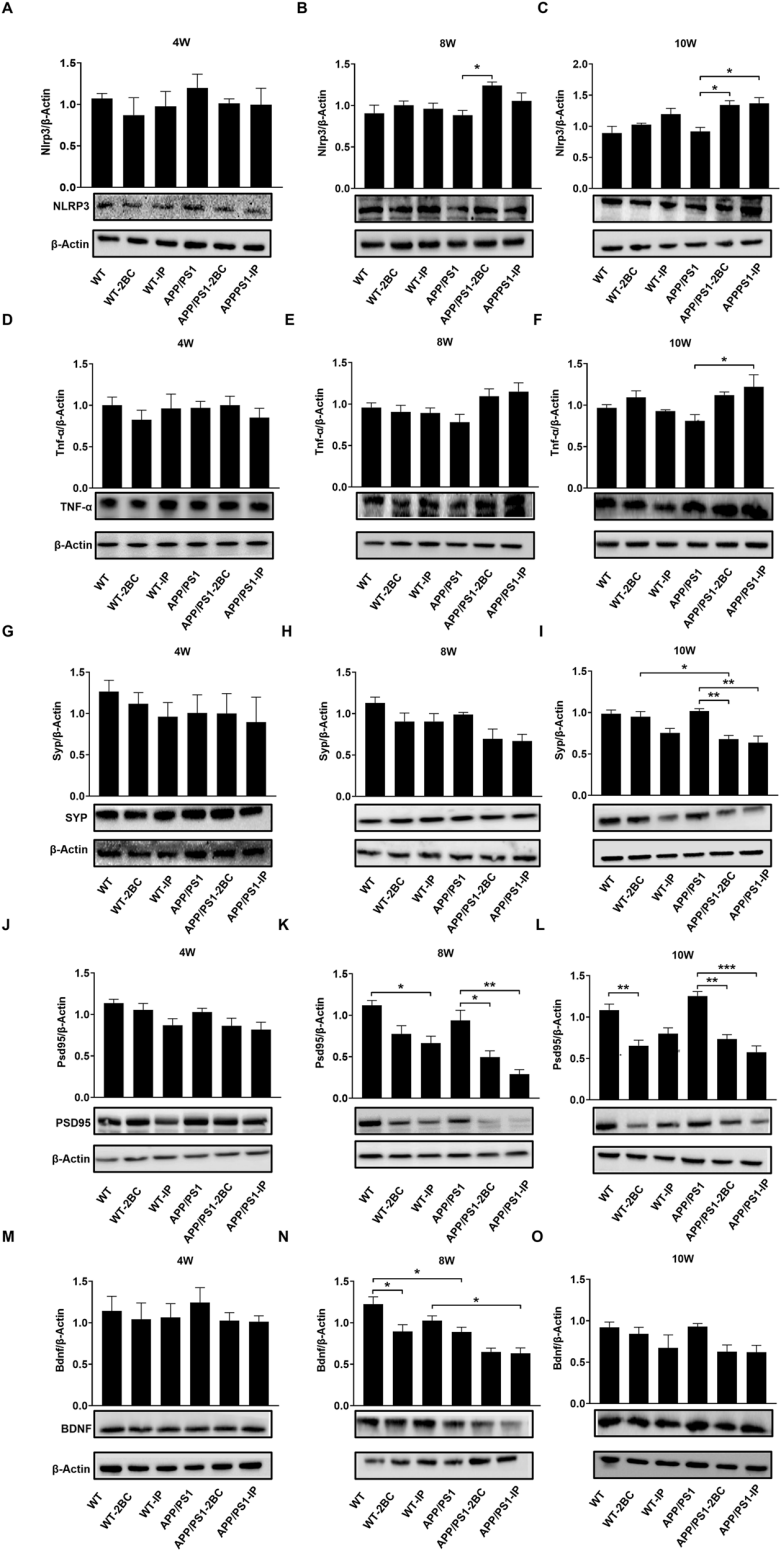
Alteration of inflammatory factors and pivotal proteins involved in dendritic and synaptic development in the ethanol-exposed WT and APP/PS1 mice. Samples of hippocampus of 8-week and 10-week ethanol-exposed WT and APP/PS1 mice were collected, western blot was conducted to detect the inflammatory factors NLRP3 (**A**–**C**) and TNF-α (**D–F**), Meanwhile, pivotal proteins involved in dendritic and synaptic development including SYP (**G**–**I**), PSD95 (**J**–**L**), and BDNF (**M**–**O**) were also detected. The relative expression of protein levels was calculated using ImageJ software. Each group contained three mice, *p < 0.05, **p < 0.01, ***p < 0.001.

To further explore the underlying mechanisms of ethanol-induced neuroinflammatory impairments, we firstly detected the protein expression level of crucial inflammatory factors, including NLRP3 and TNF-α. Elevated NLRP3 and TNF-α protein levels were observed in the hippocampus of ethanol exposed APP/PS1 mice, and no significant change was observed in ethanol-exposed WT mice (Fig. 4A–F). Neuroinflammation and oxidative stress are commonly concomitant with neurodevelopmental impairments. Next, we also examined the pivotal proteins involved in dendritic and synaptic development, such as SYP, PSD95, and BDNF in the hippocampus of ethanol-exposed mice. Earlier and more pronounced reductions of SYP, PSD95, and BDNF were observed in the hippocampus of ethanol-exposed APP/PS1 mice compared to WT mice (Fig. 4G–O). Taken together, our results showed exacerbated deteriorating neuroinflammatory impairments induced by ethanol exposure to APP/PS1 mice compared to WT mice at molecular levels.

### NAC may ameliorate the cognitive behavioral impairments induced by chronic ethanol exposure in APP/PS1 mice

**Fig. 5:**
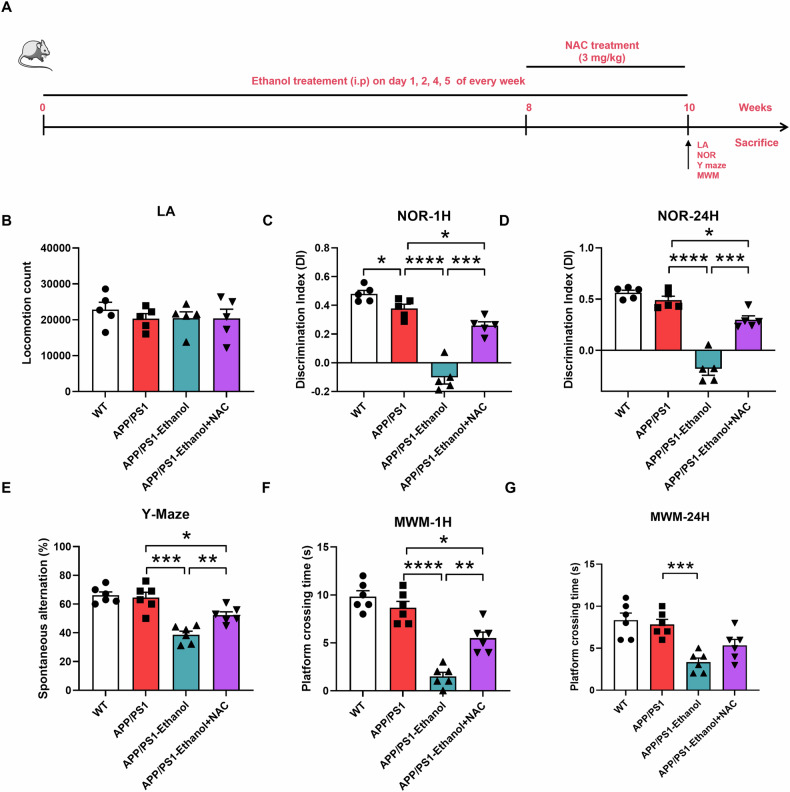
NAC partially restored cognitive behavioral impairments in ethanol-exposed APP/PS1 mice. **A** The schematic illustration of 10-week ethanol exposure process (i.p.) in APP/PS1 mice. Mice were treated with NAC (3 mg/kg) after 8-week ethanol exposure for two weeks (**B**) Locomotor activity assessments of APP/PS1 mice in LA test after 10-week ethanol exposure, the spontaneous locomotion activity was detected, no significant difference was found. The NOR tests(**C**), (**D**), Y maze tests (**E**) and MWM tests (**F**), (**G**) were performed to evaluate the cognitive behavioral alteration of APP/PS1 mice after 10-week ethanol exposure and NAC co-treatment. The cognitive behavioral declines were found in ethanol exposure group and can be partially restored after NAC co-treatment in APP/PS1 mice. Each group contains five to six mice. *p < 0.05, **p < 0.01, ***p < 0.001, ****p < 0.0001, *n* = 5 or 6.

To explore the approach for ameliorating cognitive behavioral impairments induced by ethanol exposure in APP/PS1 mice, N-acetylcysteine (NAC), a well-known antioxidant was employed (Fig. 5A). Firstly, the LA test was performed to rule out the influence of NAC on motor nerve injury. Results showed no significant change on the LA test after ethanol exposure or NAC co-treatment (Fig. 5B). Meanwhile, in the NOR, Y maze, and MWM tests, a significant impaired performance of short-term and long-term recognition memory, spatial working memory, and spatial reference memory was observed in 10-week ethanol-exposed APP/PS1 mice, while NAC co-treatment may partially restore the impaired cognitive behavioral performance (Fig. 5C–G and Supplementary Fig. 2), indicating the therapeutic potential of NAC in AD patients with chronic ethanol exposure.

### NAC may ameliorate the oxidative stress and neuroinflammatory impairments induced by chronic ethanol exposure in APP/PS1 mice

**Fig. 6:**
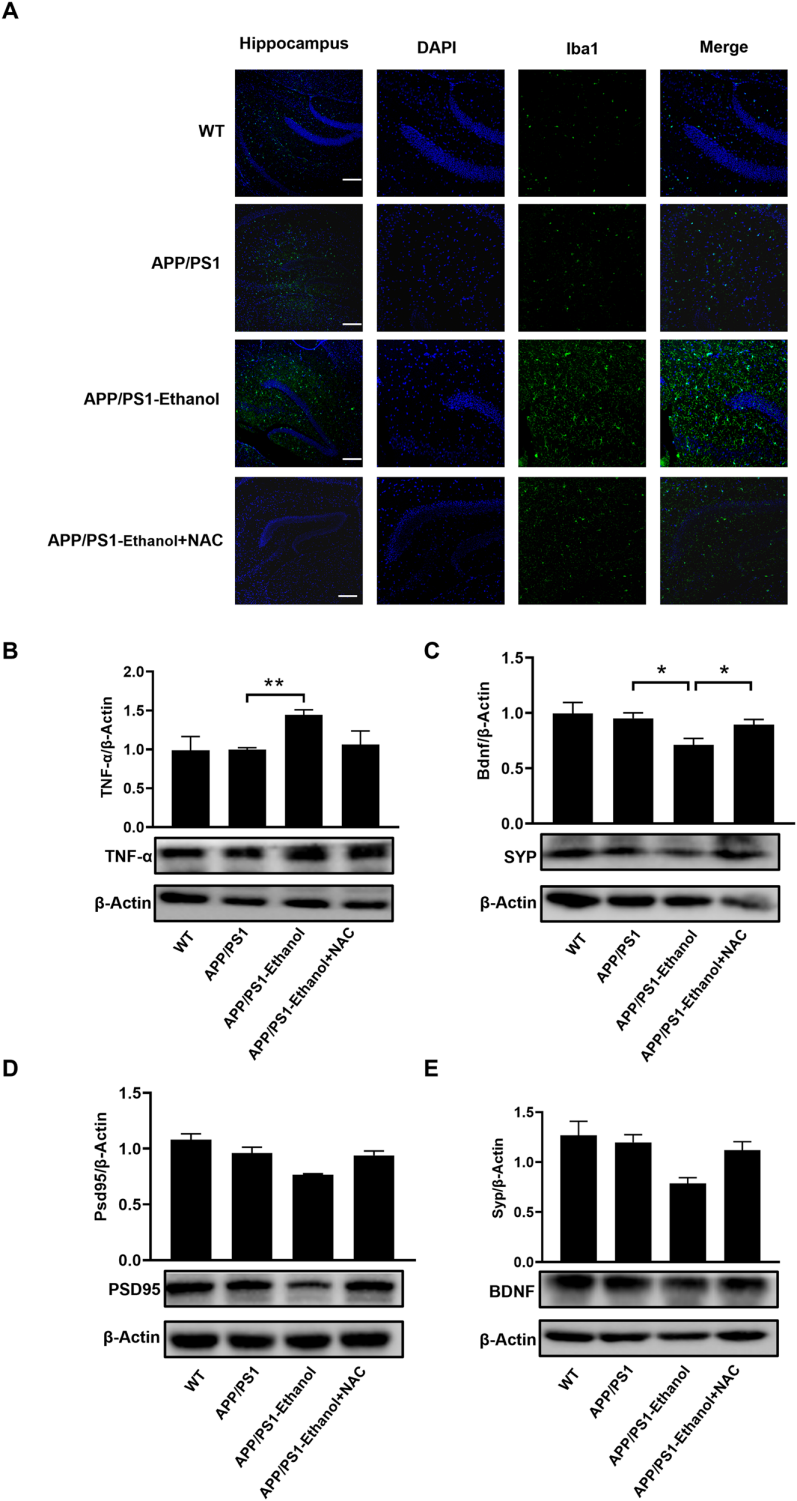
NAC treatment may partially restore ethanol exposure induced neuroinflammatory impairments in APP/PS1 mice. **A** Representative images of Iba1 immunofluorescence results in the 10-week ethanol-exposed mice. And the Iba1^+^ was increased in ethanol exposure group in APP/PS1 mice and can be partially restored after NAC co-treatment. Western blot revealed that the elevated TNF-α (**B**), and reduced SYP (**C**), PSD95 (**D**), and BDNF (**E**) protein levels induced by ethanol exposure in APP/PS1 mice can be partially restored after NAC co-treatment. Each group contains three mice, *p < 0.05, **p < 0.01.

To further explore the effect of NAC on alleviating oxidative stress and neuroinflammatory damage induced by ethanol exposure in APP/PS1 mice, we then detected the activation of microglia. Results showed that ethanol exposure markedly elevated the number of Iba1^+^ cells in the hippocampus of APP/PS1 mice, while NAC co-treatment may partially ameliorate the elevated number of Iba1^+^ cells in the hippocampus of ethanol-exposed APP/PS1 mice (Fig. 6A). Moreover, an up-regulated TNF-α protein level and decreased SYP, PSD95 and BDNF protein level was observed in the hippocampus of ethanol-exposed APP/PS1 mice, while the altered protein level was partially restored after being treated with NAC (Fig. 6B–E). Taken together, we have demonstrated that NAC may ameliorate oxidative stress and neuroinflammatory impairments induced by chronic ethanol exposure in APP/PS1 mice.

## Discussion

Chronic ethanol exposure is recognized as a risk factor of AD and associated with the development of cognitive behavioral deficits [27, 28]. However, the precise relationship between AD and chronic ethanol exposure remains to be fully elucidated. In this research, we adopted three ethanol exposure time points to track the effects of ethanol exposure on cognitive behavior. Four different ethanol exposure paradigms were employed to simulate the various drinking pattern observed in humans. Our results showed a 10-week chronic ethanol exposure in all four drinking paradigms was sufficient to cause significant cognitive behavioral impairments in both WT and APP/PS1 mice during NOR, Y-maze, and MWM tests. And earlier and severer cognitive behavioral impairments were observed in chronic ethanol-exposed APP/PS1 mice, indicating that chronic ethanol exposure may exacerbate AD pathology and thereby affect the cognitive behavioral impairments.

Aβ is a crucial biomarker of AD and plays a critical role in the initiation and progression of AD [29]. Studies have indicated that high ethanol exposure may increase the level of APP and beta-site APP cleaving enzyme 1 (BACE1) thus significantly promoted Aβ production both in vitro and in vivo [6]. Recent research found that a 10-week ethanol exposure in 2BC paradigm may cause a greater number of smaller amyloid plaques in the cortex and hippocampus [30]. In our study, we found that the increased Aβ plaques firstly appeared in the cortex and hippocampus of earlier 8-week ethanol exposed APP/PS1 mice (in both 2BC and IP paradigms), and no Aβ plaque was found even after 10 weeks’ ethanol exposure in WT mice, suggesting that chronic ethanol exposure did not directly result in Aβ plaques but accelerate the Aβ deposition. Furthermore, chronic ethanol exposure may exacerbate impairments to AD patients.

Aβ has also been demonstrated to activate microglia and result in inflammatory damages [11]. Microglia, as the immune defender of the central nervous system (CNS), plays a crucial role in numerous neurodegenerative diseases. In the process of chronic inflammation, microglia are activated, and then a series of inflammatory mediators are continuously released, leading to oxidative stress response. This sustained inflammatory response is believed to contribute to neuronal damage [31, 32]. In our study, we found microglia activation in the hippocampus of APP/PS1 mice after ethanol exposure. And with the increased time of ethanol intake, more activated microglia were found, suggesting the continuous elevated oxidative stress and neuroinflammatory damage induced by ethanol exposure. And the earlier and greater number of microglia activation was found in APP/PS1 mice compared to WT mice, further suggesting ethanol exposure may cause more neuroinflammatory damage in APP/PS1 mice. Oxidative stress is a pathological state in which the production of oxygen free radicals in the body exceeds the body’s antioxidant capacity. It is considered an critical factor leading to aging and diseases [33]. In our study, an earlier and greater decline of T-AOC was found in the hippocampal of APP/PS1 mice compared to WT mice, indicating the exacerbated decline of antioxidant capacity in APP/PS1 mice. This phenomenon could be attributed to the fact that ethanol exposure accelerated the pathological process of AD, which in turn damaged the antioxidant capacity in APP/PS1 mice. ATP is the principal energy currency of the cell, which maintains a pivotal relationship with oxidative stress and serves as a critical indicator of cellular metabolic health and redox balance. Studies have shown that ATP synthase activity and ATP level were reduced in the brains of AD patients [34, 35]. In our study, a significant decline of ATP level was found in the hippocampal of both WT and APP/PS1 mice after ethanol exposure, and a greater decline of ATP level was observed in APP/PS1 mice compared to WT mice. In general, our results showed that ethanol exposure may reduce the antioxidant capacity in the hippocampal of WT mice, leading to the occurrence of neuroinflammation, while in APP/PS1 mice, ethanol exposure further worsened this process by exacerbating the pathological progression of AD.

Neuroinflammation is a critical pathological phenotype of AD, and many studies indicate that NLRP3 and TNF-α play great roles in neuroinflammation of AD pathology [36, 37]. Our results showed that ethanol exposure for 8–10 weeks may activate the inflammasome NLRP3, promoting the secretion of the pro-inflammatory factor TNF-α in the hippocampus of ethanol-exposed APP/PS1 mice, which is consistent with the results of Aβ depositions and impaired antioxidant capacity. Excessive aggregation of Aβ can excessively induce inflammatory responses, further damaging neurons and synapses, ultimately leading to widespread brain damage and cognitive impairment [38]. PSD95, the most abundant scaffold protein within the cytoskeleton, which forms the structural foundation essential for synaptic plasticity. Meanwhile, SYP—a presynaptic vesicle marker—is widely utilized in studies on synaptic damage and regeneration. PSD95 and SYP are postsynaptic and presynaptic markers, respectively, used as indicators of synaptic quantity in AD mouse models [39, 40]. BDNF is widely present in the brain and participates in the growth, differentiation, and survival of neuronal cells, and its expression level is closely related to memory function [41, 42]. In our study, it was found that ethanol exposure resulted in significant loss of synaptic markers SYP and PSD95, as well as decrease in BDNF levels in the hippocampus of APP/PS1 mice, indicating the accumulated neural damages caused by chronic ethanol exposure at molecular level. Consistently, the decline of SYP, PSD95, and BDNF protein occurred earlier and with greater severity in APP/PS1 mice compared to WT mice, indicating that ethanol exposure may cause more neuroinflammatory damage in APP/PS1 mice at molecular level.

Glutathione (GSH) plays a crucial role in maintaining cellular antioxidant function, and NAC can minimize ROS oxidation by preventing GSH depletion [20, 21]. It has been reported that the level of lipid peroxides in the temporal lobe and cerebral cortex of AD patients was increased, compared to the age-matched control group [5]. Previous studies have shown that there is a decrease in glutathione and metabolic abnormalities in the cortical regions and hippocampus of AD patients [43, 44]. In our study, NAC co-treatment not only ameliorated the impaired performance in NOR, Y maze, and MWM in chronic ethanol-exposed APP/PS1 mice, but also significantly alleviated the neuroinflammation, downregulated TNF-α protein level and restored SYP, PSD95 and BDNF protein levels in the hippocampus of ethanol-exposed APP/PS1 mice, further suggesting the critical role of chronic ethanol exposure mediated oxidative stress and neuroinflammatory damage in AD pathology and the cognitive behavioral impairments.

## Conclusions

In general, our study indicated that chronic ethanol exposure may cause exacerbated continuously deteriorating cognitive impairment and neuroinflammatory damage in APP/PS1 mice compared to WT mice. Besides, we demonstrated the shared mechanistic connections between chronic ethanol exposure and AD: oxidative stress and neuroinflammation, proved that chronic ethanol exposure may accelerate AD pathology by increasing Aβ aggregation in mice and causing oxidative stress and neuroinflammatory impairments. Moreover, antioxidant NAC may improve cognitive behavioral disorders in APP/PS1 mice induced by ethanol exposure by reducing oxidative stress and neuroinflammatory impairments in the brain of mouse.

## Supplementary information

Supplementary file

## Data Availability

All data generated or analyzed during this study are included in this published article.

## References

[1] Ji Q, Chen J, Li Y, Tao E, Zhan Y. Incidence and prevalence of Alzheimer’s disease in China: a systematic review and meta-analysis. Eur J Epidemiol. 2024;39:701–14. 10.1007/s10654-024-01144-2 39088069 10.1007/s10654-024-01144-2

[2] Anitha K, Singh MK, Kohat K, Sri Varshini T, Chenchula S, Padmavathi R, et al. Recent insights into the neurobiology of Alzheimer’s disease and advanced treatment strategies. Mol Neurobiol. 2024;62:2314–32. 10.1007/s12035-024-04384-1 39102108 10.1007/s12035-024-04384-1

[3] Saroja SR, Sharma A, Hof PR, Pereira AC. Differential expression of tau species and the association with cognitive decline and synaptic loss in Alzheimer’s disease. Alzheimers Dement. 2022;18:1602–15. 10.1002/alz.12518 34873815 10.1002/alz.12518 PMC9170833

[4] Hou Y, Dan X, Babbar M, Wei Y, Hasselbalch SG, Croteau DL, et al. Ageing as a risk factor for neurodegenerative disease. Nat Rev Neurol. 2019;15:565–81. 10.1038/s41582-019-0244-7 31501588 10.1038/s41582-019-0244-7

[5] DiCiero Miranda M, de Bruin VM, Vale MR, Viana GS. Lipid peroxidation and nitrite plus nitrate levels in brain tissue from patients with Alzheimer’s disease. Gerontology. 2000;46:179–84. 10.1159/000022156 10859455 10.1159/000022156

[6] Huang D, Yu M, Yang S, Lou D, Zhou W, Zheng L, et al. Ethanol alters APP processing and aggravates alzheimer-associated phenotypes. Mol Neurobiol. 2018;55:5006–18. 10.1007/s12035-017-0703-3 28799137 10.1007/s12035-017-0703-3

[7] Harper C. The neuropathology of alcohol-related brain damage. Alcohol Alcohol. 2009;44:136–40. 10.1093/alcalc/agn102 19147798 10.1093/alcalc/agn102

[8] Flanigan MR, Royse SK, Cenkner DP, Kozinski KM, Stoughton CJ, Himes ML, et al. Imaging beta-amyloid (Abeta) burden in the brains of middle-aged individuals with alcohol-use disorders: a [(11)C]PIB PET study. Transl Psychiatry. 2021;11:257. 10.1038/s41398-021-01374-y 33934110 10.1038/s41398-021-01374-y PMC8088438

[9] Tolar M, Abushakra S, Sabbagh M. The path forward in Alzheimer’s disease therapeutics: reevaluating the amyloid cascade hypothesis. Alzheimers Dement. 2020;16:1553–60. 10.1016/j.jalz.2019.09.075 31706733 10.1016/j.jalz.2019.09.075

[10] Tang Y, Le W. Differential roles of M1 and M2 microglia in neurodegenerative diseases. Mol Neurobiol. 2016;53:1181–94. 10.1007/s12035-014-9070-5 25598354 10.1007/s12035-014-9070-5

[11] Leng F, Edison P. Neuroinflammation and microglial activation in Alzheimer disease: where do we go from here? Nat Rev Neurol. 2021;17:157–72. 10.1038/s41582-020-00435-y 33318676 10.1038/s41582-020-00435-y

[12] Keren-Shaul H, Spinrad A, Weiner A, Matcovitch-Natan O, Dvir-Szternfeld R, Ulland TK, et al. A unique microglia type associated with restricting development of Alzheimer’s disease. Cell. 2017;169:1276–90.e17. 10.1016/j.cell.2017.05.018 28602351 10.1016/j.cell.2017.05.018

[13] McManus RM, Latz E. NLRP3 inflammasome signalling in Alzheimer’s disease. Neuropharmacology. 2024;252:109941. 10.1016/j.neuropharm.2024.109941 38565393 10.1016/j.neuropharm.2024.109941

[14] Liu L, Liu W, Sun Y, Dong X. Serum albumin-embedding copper nanoclusters inhibit Alzheimer’s beta-amyloid fibrillogenesis and neuroinflammation. J Colloid Interface Sci. 2024;672:53–62. 10.1016/j.jcis.2024.05.193 38830318 10.1016/j.jcis.2024.05.193

[15] Rodrigues MES, Bolen ML, Blackmer-Raynolds L, Schwartz N, Chang J, Tansey MG, et al. Diet-induced metabolic and immune impairments are sex-specifically modulated by soluble TNF signaling in the 5xFAD mouse model of Alzheimer’s disease. Neurobiol Dis. 2024;196:106511. 10.1016/j.nbd.2024.106511 38670277 10.1016/j.nbd.2024.106511

[16] Fernandez-Morales JC, Arranz-Tagarro JA, Calvo-Gallardo E, Maroto M, Padin JF, Garcia AG. Stabilizers of neuronal and mitochondrial calcium cycling as a strategy for developing a medicine for Alzheimer’s disease. ACS Chem Neurosci. 2012;3:873–83. 10.1021/cn3001069 23173068 10.1021/cn3001069 PMC3503342

[17] Mondragon-Rodriguez S, Perry G, Zhu X, Moreira PI, Acevedo-Aquino MC, Williams S. Phosphorylation of tau protein as the link between oxidative stress, mitochondrial dysfunction, and connectivity failure: implications for Alzheimer’s disease. Oxid Med Cell Longev. 2013;2013:940603. 10.1155/2013/940603 23936615 10.1155/2013/940603 PMC3723250

[18] Hernandez-Zimbron LF, Luna-Munoz J, Mena R, Vazquez-Ramirez R, Kubli-Garfias C, Cribbs DH, et al. Amyloid-beta peptide binds to cytochrome C oxidase subunit 1. PLoS One. 2012;7:e42344. 10.1371/journal.pone.0042344 22927926 10.1371/journal.pone.0042344 PMC3424232

[19] Schipper HM. Brain iron deposition and the free radical-mitochondrial theory of ageing. Ageing Res Rev. 2004;3:265–301. 10.1016/j.arr.2004.02.001 15231237 10.1016/j.arr.2004.02.001

[20] Aruoma OI, Halliwell B, Hoey BM, Butler J. The antioxidant action of N-acetylcysteine: its reaction with hydrogen peroxide, hydroxyl radical, superoxide, and hypochlorous acid. Free Radic Biol Med. 1989;6:593–7. 10.1016/0891-5849(89)90066-x 2546864 10.1016/0891-5849(89)90066-x

[21] Kerksick C, Willoughby D. The antioxidant role of glutathione and N-acetyl-cysteine supplements and exercise-induced oxidative stress. J Int Soc Sports Nutr. 2005;2:38–44. 10.1186/1550-2783-2-2-38 18500954 10.1186/1550-2783-2-2-38 PMC2129149

[22] Adams JD Jr., Klaidman LK, Odunze IN, Shen HC, Miller CA. Alzheimer’s and Parkinson’s disease. Brain levels of glutathione, glutathione disulfide, and vitamin E. Mol Chem Neuropathol. 1991;14:213–26. 10.1007/BF03159937 1958264 10.1007/BF03159937

[23] Markoutsa E, Xu P. Redox potential-sensitive N-Acetyl cysteine-prodrug nanoparticles inhibit the activation of microglia and improve neuronal survival. Mol Pharm. 2017;14:1591–1600. 10.1021/acs.molpharmaceut.6b01028 28335600 10.1021/acs.molpharmaceut.6b01028 PMC5534351

[24] Thiele TE, Navarro M. “Drinking in the dark” (DID) procedures: a model of binge-like ethanol drinking in non-dependent mice. Alcohol. 2014;48:235–41. 10.1016/j.alcohol.2013.08.005 24275142 10.1016/j.alcohol.2013.08.005 PMC4004717

[25] Rhodes JS, Best K, Belknap JK, Finn DA, Crabbe JC. Evaluation of a simple model of ethanol drinking to intoxication in C57BL/6J mice. Physiol Behav. 2005;84:53–63. 10.1016/j.physbeh.2004.10.007 15642607 10.1016/j.physbeh.2004.10.007

[26] Chen H, Xing H, Zhong C, Lin X, Chen R, Luo N, et al. METTL3 confers protection against mitochondrial dysfunction and cognitive impairment in an Alzheimer disease mouse model by upregulating Mfn2 via N6-methyladenosine modification. J Neuropathol Exp Neurol. 2024;83:606–14. 10.1093/jnen/nlae010 38408379 10.1093/jnen/nlae010

[27] Xie C, Feng Y. Alcohol consumption and risk of Alzheimer’s disease: a dose-response meta-analysis. Geriatr Gerontol Int. 2022;22:278–85. 10.1111/ggi.14357 35171516 10.1111/ggi.14357

[28] Sun M, Zheng Q, Wang L, Wang R, Cui H, Zhang X, et al. Alcohol consumption during adolescence alters the cognitive function in adult male mice by persistently increasing levels of DUSP6. Mol Neurobiol. 2024;61:3161–78. 10.1007/s12035-023-03794-x 37978157 10.1007/s12035-023-03794-x

[29] Xia Y, Dore V, Fripp J, Bourgeat P, Laws SM, Fowler CJ, et al. Association of basal forebrain atrophy with cognitive decline in early Alzheimer disease. Neurology. 2024;103:e209626. 10.1212/WNL.0000000000209626 38885444 10.1212/WNL.0000000000209626 PMC11254448

[30] Day SM, Gironda SC, Clarke CW, Snipes JA, Nicol NI, Kamran H, et al. Ethanol exposure alters Alzheimer’s-related pathology, behavior, and metabolism in APP/PS1 mice. Neurobiol Dis. 2023;177:105967. 10.1016/j.nbd.2022.105967 36535550 10.1016/j.nbd.2022.105967 PMC10010148

[31] Kapasi A, Yu L, Leurgans SE, Agrawal S, Boyle PA, Bennett DA, et al. Association between hippocampal microglia, AD and LATE-NC, and cognitive decline in older adults. Alzheimers Dement. 2024;20:3193–202. 10.1002/alz.13780 38494787 10.1002/alz.13780 PMC11095444

[32] Wood H. alpha-Synuclein-activated microglia are implicated in PD pathogenesis. Nat Rev Neurol. 2022;18:188. 10.1038/s41582-022-00631-y 35165429 10.1038/s41582-022-00631-y

[33] Chen JX, Yan SD. Amyloid-beta-induced mitochondrial dysfunction. J Alzheimers Dis. 2007;12:177–84. 10.3233/jad-2007-12208 17917162 10.3233/jad-2007-12208 PMC3687350

[34] Terni B, Boada J, Portero-Otin M, Pamplona R, Ferrer I. Mitochondrial ATP-synthase in the entorhinal cortex is a target of oxidative stress at stages I/II of Alzheimer’s disease pathology. Brain Pathol. 2010;20:222–33. 10.1111/j.1750-3639.2009.00266.x 19298596 10.1111/j.1750-3639.2009.00266.x PMC8094794

[35] Cha MY, Cho HJ, Kim C, Jung YO, Kang MJ, Murray ME, et al. Mitochondrial ATP synthase activity is impaired by suppressed O-GlcNAcylation in Alzheimer’s disease. Hum Mol Genet. 2015;24:6492–504. 10.1093/hmg/ddv358 26358770 10.1093/hmg/ddv358 PMC5007609

[36] Wang SY, Fu XX, Duan R, Wei B, Cao HM, Yan E, et al. The Alzheimer’s disease-associated gene TREML2 modulates inflammation by regulating microglia polarization and NLRP3 inflammasome activation. Neural Regen Res. 2023;18:434–8. 10.4103/1673-5374.346468 35900442 10.4103/1673-5374.346468 PMC9396521

[37] Sun J, Zhang Y, Kong Y, Ye T, Yu Q, Kumaran Satyanarayanan S, et al. Microbiota-derived metabolite Indoles induced aryl hydrocarbon receptor activation and inhibited neuroinflammation in APP/PS1 mice. Brain Behav Immun. 2022;106:76–88. 10.1016/j.bbi.2022.08.003 35961580 10.1016/j.bbi.2022.08.003

[38] Sun XY, Yu XL, Zhu J, Li LJ, Zhang L, Huang YR, et al. Fc effector of anti-Abeta antibody induces synapse loss and cognitive deficits in Alzheimer’s disease-like mouse model. Signal Transduct Target Ther. 2023;8:30. 10.1038/s41392-022-01273-8 36693826 10.1038/s41392-022-01273-8 PMC9873795

[39] Guo L, Yang X, Zhang Y, Xu X, Li Y. Effect of exercise on cognitive function and synaptic plasticity in Alzheimer’s disease models: a systematic review and meta-analysis. Front Aging Neurosci. 2022;14:1077732. 10.3389/fnagi.2022.1077732 36704501 10.3389/fnagi.2022.1077732 PMC9872519

[40] Shao CY, Mirra SS, Sait HB, Sacktor TC, Sigurdsson EM. Postsynaptic degeneration as revealed by PSD-95 reduction occurs after advanced Abeta and tau pathology in transgenic mouse models of Alzheimer’s disease. Acta Neuropathol. 2011;122:285–92. 10.1007/s00401-011-0843-x 21630115 10.1007/s00401-011-0843-x PMC3437675

[41] Zhao M, Chen X, Liu J, Feng Y, Wang C, Xu T. et al. Sorl1 knockout inhibits expression of brain-derived neurotrophic factor: involvement in the development of late-onset Alzheimeras disease. Neural Regen Res. 2024;19:1602–7. 38051905 10.4103/1673-5374.387975 PMC10883503

[42] You S, Ma Z, Zhang P, Xu W, Zhan C, Sang N, et al. Neuroprotective effects of the salidroside derivative SHPL-49 via the BDNF/TrkB/Gap43 pathway in rats with cerebral ischemia. Biomed Pharmacother. 2024;174:116460. 10.1016/j.biopha.2024.116460 38520864 10.1016/j.biopha.2024.116460

[43] Jain SK, Stevens CM, Margret JJ, Levine SN. Alzheimer’s disease: a review of pathology, current treatments, and the potential therapeutic effect of decreasing oxidative stress by combined vitamin D and l-cysteine supplementation. Antioxid Redox Signal. 2024;40:663–78. 10.1089/ars.2023.0245 37756366 10.1089/ars.2023.0245 PMC11001507

[44] Martinez-Banaclocha M. N-Acetyl-Cysteine: modulating the cysteine redox proteome in neurodegenerative diseases. Antioxidants. 2022;11:416. 10.3390/antiox11020416 35204298 10.3390/antiox11020416 PMC8869501

