# Supplementary material for: N-acetylcysteine (NAC) ameliorates ethanol-induced oxidative stress, neuroinflammation, and cognitive dysfunction in APP/PS1 mouse model
Source: Transl Psychiatry. 2025 Oct 24;15:435. doi: 10.1038/s41398-025-03496-z (PMC12552700; doi:10.1038/s41398-025-03496-z)
Supplement: Supplementary file 1 — Supplementary file [file 41398_2025_3496_MOESM1_ESM.pdf]

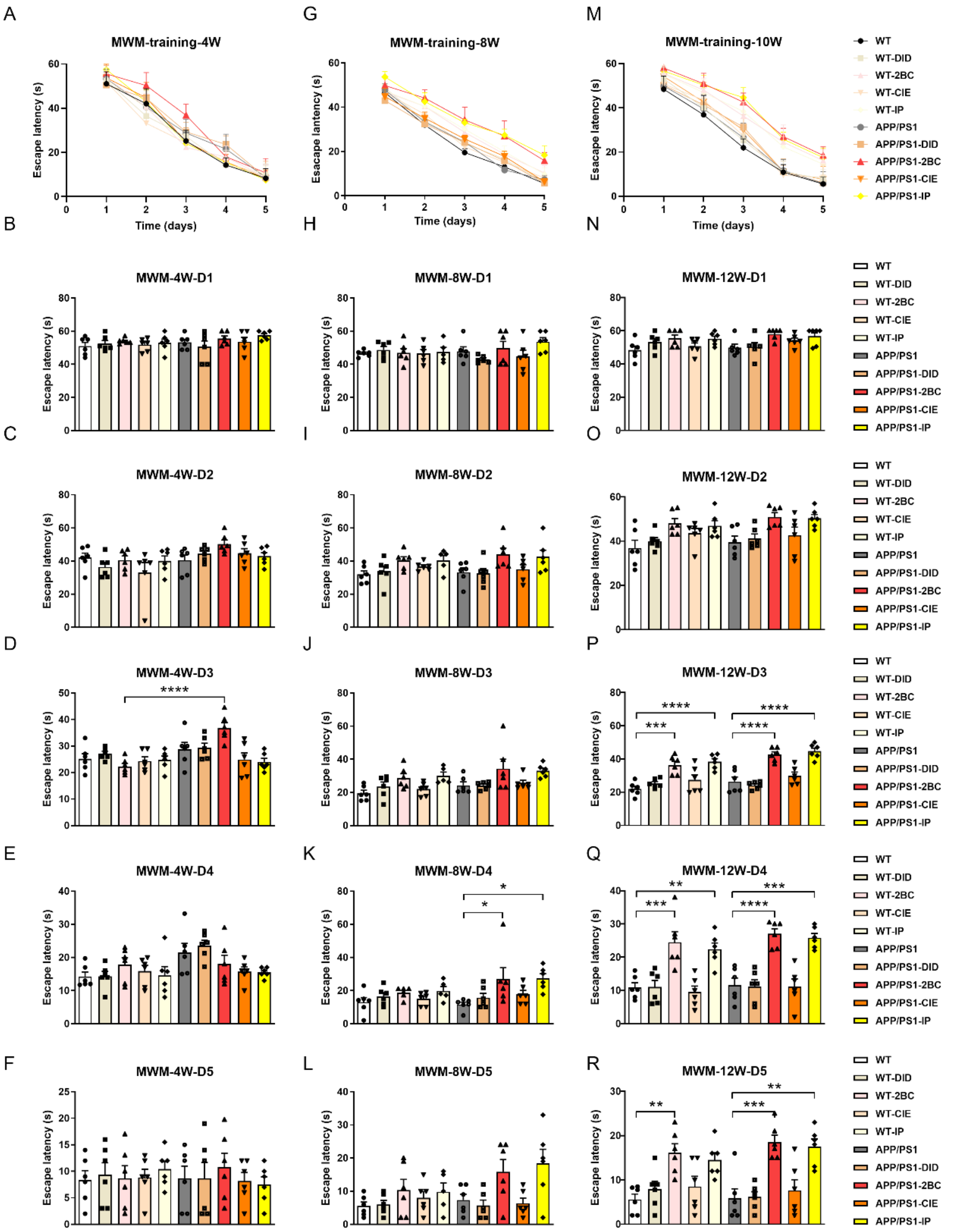

Supplementary figure1: The escape latency in the training stage of the MWM which were performed to examine spatial reference memory of WT and APP/PS1 mice after 4-week (A-F), 8-week (G-L), and 10-week (M-R) of ethanol exposure. The line chart aggregates data across all groups, while the bar chart illustrates the statistical differences among groups for each respective day from Day 1 to Day 5 (D1-D5). *$p < 0.05$, **$p < 0.01$, ***$p < 0.001$, ****$p < 0.0001$.

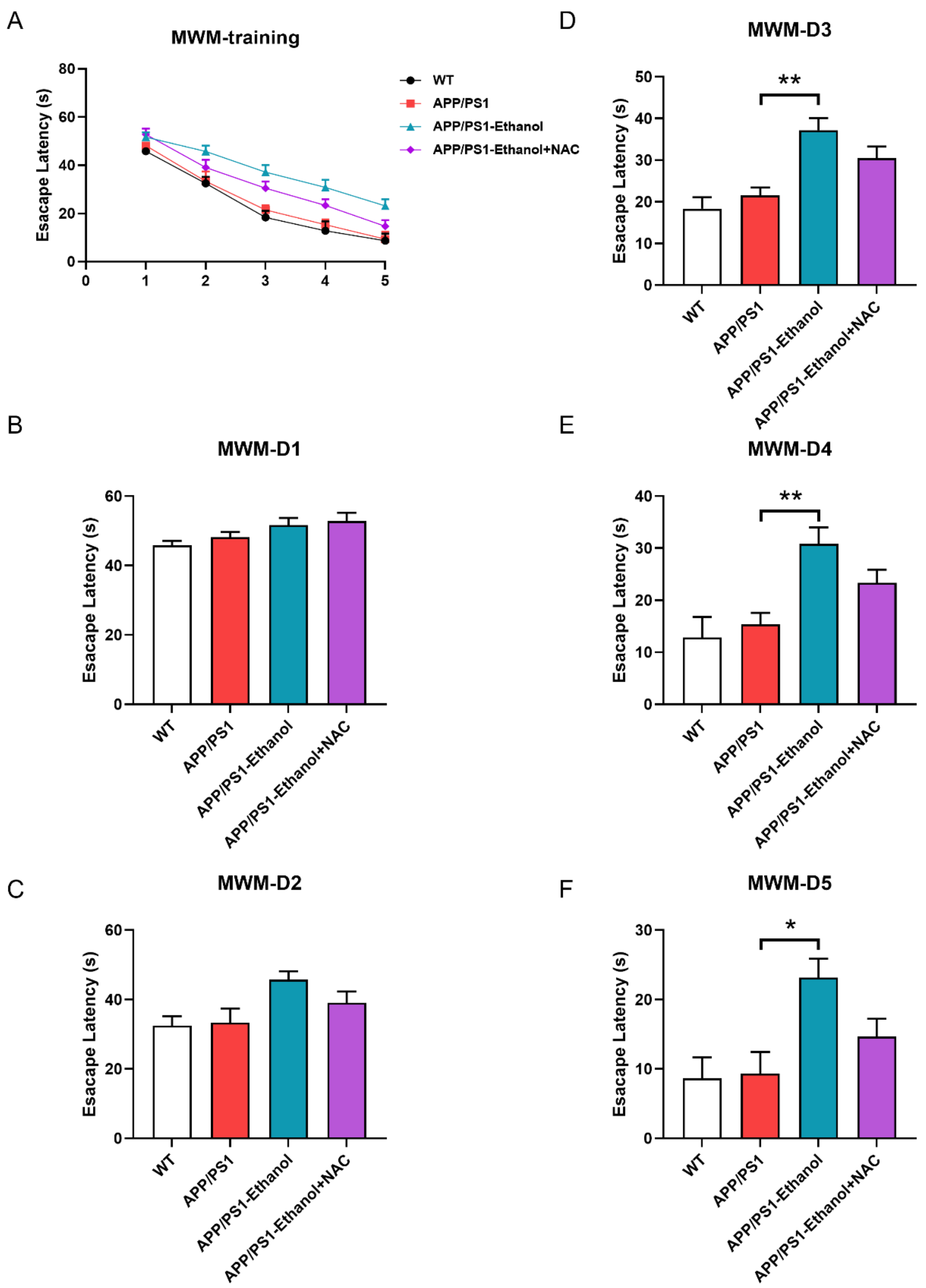

Supplementary figure2: The escape latency in the training stage of the MWM tests (A-F) were performed to evaluate the cognitive behavioral alteration of APP/PS1 mice after 10-week of ethanol exposure and NAC co-treatment. *$p < 0.05$, **$p < 0.01$.

Supplementary Table 1

Figure1					
**figure1**	**F**			**G**	
		P	t/q	P	t/q
n			5~6		5~6
interaction	**	P=0.0015	F (4, 46) = 5.227		
row factor	****	P<0.0001	F (4, 46) = 14.73		
column factor	****	P<0.0001	F (1, 46) = 28.29		
method	two way ANOVA			kolmogorov-Smirnov test	
	Tukey				
NC:WT vs. NC:APP	ns	>0.9999	0.6377	0.85449	0.5
NC:WT vs. DID:WT	ns	0.9998	0.835	0.046602	1
NC:WT vs. DID:APP	ns	0.2197	3.765		
NC:WT vs. 2BC:WT	ns	0.5504	2.941	0.027776	1
NC:WT vs. 2BC:APP	***	0.0003	7.186		
NC:WT vs. CIE:WT	ns	0.9991	1.032	0.046602	1
NC:WT vs. CIE:APP	ns	0.9578	1.78		
NC:WT vs. IP:WT	ns	0.6936	2.632	0.046602	1
NC:WT vs. IP:APP	****	<0.0001	11.59		
NC:APP vs. DID:WT	ns	>0.9999	0.227		
NC:APP vs. DID:APP	ns	0.4509	3.157	0.168248	0.8333
NC:APP vs. 2BC:WT	ns	0.8272	2.303		
NC:APP vs. 2BC:APP	**	0.0011	6.549	0.168248	0.8333
NC:APP vs. CIE:WT	ns	>0.9999	0.424		
NC:APP vs. CIE:APP	ns	0.9981	1.142	0.460225	0.6667
NC:APP vs. IP:WT	ns	0.9107	2.024		
NC:APP vs. IP:APP	****	<0.0001	10.95	0.027776	1
DID:WT vs. DID:APP	ns	0.6144	2.805	0.061756	1
DID:WT vs. 2BC:WT	ns	0.9234	1.969		
DID:WT vs. 2BC:APP	**	0.0037	6.017		
DID:WT vs. CIE:WT	ns	>0.9999	0.1886		
DID:WT vs. CIE:APP	ns	0.9998	0.8619		
DID:WT vs. IP:WT	ns	0.9658	1.721		
DID:WT vs. IP:APP	****	<0.0001	10.21		
DID:APP vs. 2BC:WT	ns	0.9995	0.9604		
DID:APP vs. 2BC:APP	ns	0.4822	3.087		
DID:APP vs. CIE:WT	ns	0.7009	2.616		
DID:APP vs. CIE:APP	ns	0.8998	2.068		
DID:APP vs. IP:WT	ns	0.9987	1.084		
DID:APP vs. IP:APP	***	0.0002	7.283		
2BC:WT vs. 2BC:APP	ns	0.1078	4.245	0.930736	0.3333
2BC:WT vs. CIE:WT	ns	0.9589	1.772		
2BC:WT vs. CIE:APP	ns	0.9979	1.161		
2BC:WT vs. IP:WT	ns	>0.9999	0.1717		
2BC:WT vs. IP:APP	****	<0.0001	8.645		
2BC:APP vs. CIE:WT	**	0.0056	5.82		
2BC:APP vs. CIE:APP	*	0.0131	5.407		
2BC:APP vs. IP:WT	ns	0.1124	4.219		
2BC:APP vs. IP:APP	ns	0.0838	4.4		
CIE:WT vs. CIE:APP	ns	>0.9999	0.6649	0.168248	0.8333
CIE:WT vs. IP:WT	ns	0.984	1.532		
CIE:WT vs. IP:APP	****	<0.0001	10.02		
CIE:APP vs. IP:WT	ns	0.9996	0.9355		
CIE:APP vs. IP:APP	****	<0.0001	9.807		
IP:WT vs. IP:APP	****	<0.0001	8.415	0.90285	0.4

figure1	J			M		
		P	t/q		P	t/q
n			5~6			6
interaction	ns	P=0.2946	F (4, 46) = 1.272	ns	P=0.8342	F (4, 50) = 0.3624
row factor	****	P<0.0001	F (4, 46) = 22.35	****	P<0.0001	F (4, 50) = 10.03
column factor	ns	P=0.8374	F (1, 46) = 0.0426[illegible]	*	P=0.0481	F (1, 50) = 4.104
method	two way ANOVA			two way ANOVA		
	Tukey			Tukey		
NC:WT vs. NC:APP	ns	0.9996	0.9312	ns	>0.9999	0.3864
NC:WT vs. DID:WT	ns	0.9341	1.918	ns	0.4511	3.153
NC:WT vs. DID:APP	ns	0.7187	2.576	ns	0.1769	3.91
NC:WT vs. 2BC:WT	****	<0.0001	8.915	*	0.0182	5.214
NC:WT vs. 2BC:APP	***	0.0005	6.902	***	0.0007	6.685
NC:WT vs. CIE:WT	ns	0.1869	3.881	ns	0.2948	3.532
NC:WT vs. CIE:APP	*	0.0414	4.804	*	0.0476	4.709
NC:WT vs. IP:WT	*	0.0116	5.465	ns	0.1563	3.994
NC:WT vs. IP:APP	****	<0.0001	7.93	***	0.0009	6.608
NC:APP vs. DID:WT	ns	0.6139	2.806	ns	0.6319	2.767
NC:APP vs. DID:APP	ns	0.3223	3.464	ns	0.2978	3.524
NC:APP vs. 2BC:WT	****	<0.0001	9.846	*	0.0383	4.827
NC:APP vs. 2BC:APP	****	<0.0001	7.833	**	0.0018	6.299
NC:APP vs. CIE:WT	*	0.0442	4.769	ns	0.4547	3.145
NC:APP vs. CIE:APP	**	0.0067	5.736	ns	0.0931	4.323
NC:APP vs. IP:WT	**	0.0018	6.352	ns	0.2678	3.608
NC:APP vs. IP:APP	****	<0.0001	8.861	**	0.0021	6.222
DID:WT vs. DID:APP	ns	>0.9999	0.6296	ns	>0.9999	0.7568
DID:WT vs. 2BC:WT	**	0.0011	6.582	ns	0.9023	2.06
DID:WT vs. 2BC:APP	ns	0.0534	4.663	ns	0.2948	3.532
DID:WT vs. CIE:WT	ns	0.9415	1.879	ns	>0.9999	0.3784
DID:WT vs. CIE:APP	ns	0.6799	2.663	ns	0.9825	1.556
DID:WT vs. IP:WT	ns	0.3489	3.396	ns	0.9998	0.8409
DID:WT vs. IP:APP	**	0.0081	5.643	ns	0.3238	3.455
DID:APP vs. 2BC:WT	**	0.0045	5.924	ns	0.995	1.303
DID:APP vs. 2BC:APP	ns	0.156	4.005	ns	0.6282	2.775
DID:APP vs. CIE:WT	ns	0.9963	1.25	ns	>0.9999	0.3784
DID:APP vs. CIE:APP	ns	0.9152	2.005	ns	0.9999	0.7989
DID:APP vs. IP:WT	ns	0.6326	2.766	ns	>0.9999	0.08409
DID:APP vs. IP:APP	*	0.0297	4.985	ns	0.6639	2.698
2BC:WT vs. 2BC:APP	ns	0.9135	2.013	ns	0.988	1.472
2BC:WT vs. CIE:WT	ns	0.0577	4.619	ns	0.9707	1.682
2BC:WT vs. CIE:APP	ns	0.1332	4.11	ns	>0.9999	0.5045
2BC:WT vs. IP:WT	ns	0.5062	3.035	ns	0.9969	1.219
2BC:WT vs. IP:APP	ns	0.9994	0.9852	ns	0.9918	1.395
2BC:APP vs. CIE:WT	ns	0.663	2.7	ns	0.4511	3.153
2BC:APP vs. CIE:APP	ns	0.8918	2.098	ns	0.9224	1.976
2BC:APP vs. IP:WT	ns	0.9984	1.116	ns	0.6672	2.691
2BC:APP vs. IP:APP	ns	0.9992	1.027	ns	>0.9999	0.07694
CIE:WT vs. CIE:APP	ns	>0.9999	0.6999	ns	0.9977	1.177
CIE:WT vs. IP:WT	ns	0.9851	1.516	ns	>0.9999	0.4625
CIE:WT vs. IP:APP	ns	0.246	3.68	ns	0.4863	3.076
CIE:APP vs. IP:WT	ns	0.9998	0.8837	ns	>0.9999	0.7148
CIE:APP vs. IP:APP	ns	0.465	3.125	ns	0.9382	1.899
IP:WT vs. IP:APP	ns	0.8923	2.096	ns	0.702	2.614

figure1	R			S		
		P	t/q		P	t/q
n			6			6
interaction	ns	P=0.4375	F (4, 50) = 0.9602	ns	P=0.9046	F (4, 50) = 0.2560
row factor	***	P=0.0002	F (4, 50) = 6.852	****	P<0.0001	F (4, 50) = 11.97
column factor	**	P=0.0061	F (1, 50) = 8.205	*	P=0.0187	F (1, 50) = 5.904
method	two way ANOVA			two way ANOVA		
	Tukey			Tukey		
NC:WT vs. NC:APP	ns	>0.9999	0.2384	ns	0.9999	0.8232
NC:WT vs. DID:WT	ns	0.7981	2.384	ns	0.5133	3.019
NC:WT vs. DID:APP	ns	0.476	3.099	ns	0.1952	3.842
NC:WT vs. 2BC:WT	ns	0.7981	2.384	*	0.0182	5.214
NC:WT vs. 2BC:APP	**	0.0064	5.721	***	0.0003	7.135
NC:WT vs. CIE:WT	ns	0.9974	1.192	ns	0.5133	3.019
NC:WT vs. CIE:APP	ns	0.5879	2.86	ns	0.0515	4.665
NC:WT vs. IP:WT	ns	0.3711	3.337	*	0.0182	5.214
NC:WT vs. IP:APP	**	0.0013	6.436	****	<0.0001	7.683
NC:APP vs. DID:WT	ns	0.8789	2.145	ns	0.8638	2.195
NC:APP vs. DID:APP	ns	0.5879	2.86	ns	0.5133	3.019
NC:APP vs. 2BC:WT	ns	0.8789	2.145	ns	0.0831	4.391
NC:APP vs. 2BC:APP	*	0.0105	5.482	**	0.0017	6.311
NC:APP vs. CIE:WT	ns	0.9996	0.9535	ns	0.8638	2.195
NC:APP vs. CIE:APP	ns	0.6984	2.622	ns	0.1952	3.842
NC:APP vs. IP:WT	ns	0.476	3.099	ns	0.0831	4.391
NC:APP vs. IP:APP	**	0.0022	6.198	***	0.0005	6.86
DID:WT vs. DID:APP	ns	>0.9999	0.7151	ns	0.9999	0.8232
DID:WT vs. 2BC:WT	ns	>0.9999	0	ns	0.8638	2.195
DID:WT vs. 2BC:APP	ns	0.3711	3.337	ns	0.1297	4.116
DID:WT vs. CIE:WT	ns	0.9974	1.192	ns	>0.9999	0
DID:WT vs. CIE:APP	ns	>0.9999	0.4767	ns	0.9745	1.646
DID:WT vs. IP:WT	ns	0.9996	0.9535	ns	0.8638	2.195
DID:WT vs. IP:APP	ns	0.1432	4.052	ns	0.0515	4.665
DID:APP vs. 2BC:WT	ns	>0.9999	0.7151	ns	0.9927	1.372
DID:APP vs. 2BC:APP	ns	0.6984	2.622	ns	0.3897	3.293
DID:APP vs. CIE:WT	ns	0.9367	1.907	ns	0.9999	0.8232
DID:APP vs. CIE:APP	ns	>0.9999	0.2384	ns	0.9999	0.8232
DID:APP vs. IP:WT	ns	>0.9999	0.2384	ns	0.9927	1.372
DID:APP vs. IP:APP	ns	0.3711	3.337	ns	0.1952	3.842
2BC:WT vs. 2BC:APP	ns	0.3711	3.337	ns	0.934	1.921
2BC:WT vs. CIE:WT	ns	0.9974	1.192	ns	0.8638	2.195
2BC:WT vs. CIE:APP	ns	>0.9999	0.4767	ns	>0.9999	0.5488
2BC:WT vs. IP:WT	ns	0.9996	0.9535	ns	>0.9999	0
2BC:WT vs. IP:APP	ns	0.1432	4.052	ns	0.7639	2.47
2BC:APP vs. CIE:WT	ns	0.0655	4.529	ns	0.1297	4.116
2BC:APP vs. CIE:APP	ns	0.5879	2.86	ns	0.7639	2.47
2BC:APP vs. IP:WT	ns	0.7981	2.384	ns	0.934	1.921
2BC:APP vs. IP:APP	ns	>0.9999	0.7151	ns	>0.9999	0.5488
CIE:WT vs. CIE:APP	ns	0.9722	1.669	ns	0.9745	1.646
CIE:WT vs. IP:WT	ns	0.8789	2.145	ns	0.8638	2.195
CIE:WT vs. IP:APP	*	0.0171	5.244	ns	0.0515	4.665
CIE:APP vs. IP:WT	ns	>0.9999	0.4767	ns	>0.9999	0.5488
CIE:APP vs. IP:APP	ns	0.2791	3.575	ns	0.5133	3.019
IP:WT vs. IP:APP	ns	0.476	3.099	ns	0.7639	2.47

figure1	U			V		
		P	t/q		P	t/q
n			6		6	
interaction	ns	P=0.0573	F (4, 50) = 2.459	ns	P=0.9696	F (4, 50) = 0.1329
row factor	****	P<0.0001	F (4, 50) = 11.10	****	P<0.0001	F (4, 50) = 13.47
column factor	****	P<0.0001	F (1, 50) = 30.54	**	P=0.0017	F (1, 50) = 10.95
method	two way ANOVA				two way ANOVA	
	Tukey			Tukey		
NC:WT vs. NC:APP	ns	>0.9999	0.7825	ns	0.8002	2.378
NC:WT vs. DID:WT	ns	0.995	1.304	ns	0.2055	3.805
NC:WT vs. DID:APP	ns	0.0772	4.434	*	0.0108	5.47
NC:WT vs. 2BC:WT	ns	0.7046	2.608	***	0.0008	6.659
NC:WT vs. 2BC:APP	****	<0.0001	8.085	****	<0.0001	8.562
NC:WT vs. CIE:WT	ns	0.9511	1.826	ns	0.0997	4.281
NC:WT vs. CIE:APP	ns	0.1764	3.912	**	0.0039	5.946
NC:WT vs. IP:WT	ns	0.3492	3.391	**	0.0039	5.946
NC:WT vs. IP:APP	****	<0.0001	9.39	****	<0.0001	8.8
NC:APP vs. DID:WT	ns	>0.9999	0.5216	ns	0.9904	1.427
NC:APP vs. DID:APP	ns	0.2531	3.651	ns	0.4792	3.092
NC:APP vs. 2BC:WT	ns	0.9511	1.826	ns	0.0997	4.281
NC:APP vs. 2BC:APP	***	0.0002	7.303	**	0.0023	6.183
NC:APP vs. CIE:WT	ns	0.9991	1.043	ns	0.9376	1.903
NC:APP vs. CIE:APP	ns	0.4618	3.13	ns	0.282	3.567
NC:APP vs. IP:WT	ns	0.7046	2.608	ns	0.282	3.567
NC:APP vs. IP:APP	****	<0.0001	8.607	**	0.0014	6.421
DID:WT vs. DID:APP	ns	0.4618	3.13	ns	0.9726	1.665
DID:WT vs. 2BC:WT	ns	0.995	1.304	ns	0.591	2.854
DID:WT vs. 2BC:APP	***	0.0006	6.781	*	0.0436	4.757
DID:WT vs. CIE:WT	ns	>0.9999	0.5216	ns	>0.9999	0.4757
DID:WT vs. CIE:APP	ns	0.7046	2.608	ns	0.8804	2.14
DID:WT vs. IP:WT	ns	0.8954	2.087	ns	0.8804	2.14
DID:WT vs. IP:APP	****	<0.0001	8.085	*	0.0279	4.994
DID:APP vs. 2BC:WT	ns	0.9511	1.826	ns	0.9975	1.189
DID:APP vs. 2BC:APP	ns	0.2531	3.651	ns	0.4792	3.092
DID:APP vs. CIE:WT	ns	0.7046	2.608	ns	0.9975	1.189
DID:APP vs. CIE:APP	ns	>0.9999	0.5216	ns	>0.9999	0.4757
DID:APP vs. IP:WT	ns	0.9991	1.043	ns	>0.9999	0.4757
DID:APP vs. IP:APP	*	0.03	4.956	ns	0.3742	3.33
2BC:WT vs. 2BC:APP	*	0.0106	5.477	ns	0.9376	1.903
2BC:WT vs. CIE:WT	ns	>0.9999	0.7825	ns	0.8002	2.378
2BC:WT vs. CIE:APP	ns	0.995	1.304	ns	>0.9999	0.7135
2BC:WT vs. IP:WT	ns	>0.9999	0.7825	ns	>0.9999	0.7135
2BC:WT vs. IP:APP	***	0.0006	6.781	ns	0.8804	2.14
2BC:APP vs. CIE:WT	**	0.002	6.26	ns	0.0997	4.281
2BC:APP vs. CIE:APP	ns	0.1186	4.173	ns	0.7011	2.616
2BC:APP vs. IP:WT	*	0.0488	4.695	ns	0.7011	2.616
2BC:APP vs. IP:APP	ns	0.995	1.304	ns	>0.9999	0.2378
CIE:WT vs. CIE:APP	ns	0.8954	2.087	ns	0.9726	1.665
CIE:WT vs. IP:WT	ns	0.9818	1.565	ns	0.9726	1.665
CIE:WT vs. IP:APP	****	<0.0001	7.564	ns	0.0667	4.519
CIE:APP vs. IP:WT	ns	>0.9999	0.5216	ns	>0.9999	0
CIE:APP vs. IP:APP	*	0.0106	5.477	ns	0.591	2.854
IP:WT vs. IP:APP	**	0.0035	5.999	ns	0.591	2.018

Figure3						
figure3	E			F		
		P	t/F		P	t/F
n		3			3	
interaction	***	P=0.0003	F (2, 12) = 17.52	***	P=0.0003	F (2, 12) = 16.81
row factor	****	P<0.0001	F (2, 12) = 22.29	****	P<0.0001	F (2, 12) = 139.3
column factor	***	P=0.0002	F (1, 12) = 26.72	ns	P=0.0649	F (1, 12) = 4.128
method	two way ANOVA			two way ANOVA		
	Tukey			Tukey		
NC:WT vs. NC:APP	ns	0.6602	2.147	***	0.0008	8.348
NC:WT vs. 2BC:WT	ns	0.4296	2.732	***	0.0002	9.686
NC:WT vs. 2BC:APP	**	0.0012	7.988	****	<0.0001	11.6
NC:WT vs. IP:WT	ns	0.0975	4.178	***	0.0003	9.164
NC:WT vs. IP:APP	*	0.0237	5.376	****	<0.0001	10.62
NC:APP vs. 2BC:WT	*	0.0429	4.88	****	<0.0001	18.03
NC:APP vs. 2BC:APP	***	0.0001	10.14	****	<0.0001	19.95
NC:APP vs. IP:WT	ns	0.7068	2.031	****	<0.0001	17.51
NC:APP vs. IP:APP	**	0.0019	7.523	****	<0.0001	18.97
2BC:WT vs. 2BC:APP	*	0.0274	5.256	ns	0.7525	1.912
2BC:WT vs. IP:WT	**	0.0039	6.911	ns	0.9989	0.5222
2BC:WT vs. IP:APP	ns	0.4626	2.643	ns	0.9829	0.9373
2BC:APP vs. IP:WT	****	<0.0001	12.17	ns	0.5439	2.435
2BC:APP vs. IP:APP	ns	0.4743	2.613	ns	0.9798	0.9751
IP:WT vs. IP:APP	***	0.0002	9.554	ns	0.8982	1.46
figure3	G			H		
		P	t/F		P	t/F
n		3			3	
interaction	ns	P=0.1421	F (2, 12) = 2.305	**	P=0.0015	F (2, 12) = 11.76
row factor	****	P<0.0001	F (2, 12) = 31.06	****	P<0.0001	F (2, 12) = 69.80
column factor	*	P=0.0207	F (1, 12) = 7.086	ns	P=0.1614	F (1, 12) = 2.227
method	two way ANOVA			two way ANOVA		
	Tukey			Tukey		
NC:WT vs. NC:APP	ns	>0.9999	0.2949	ns	0.1161	4.025
NC:WT vs. 2BC:WT	*	0.0278	5.243	**	0.0053	6.646
NC:WT vs. 2BC:APP	***	0.0005	8.853	****	<0.0001	12.19
NC:WT vs. IP:WT	ns	0.0574	4.633	*	0.0285	5.221
NC:WT vs. IP:APP	**	0.0014	7.838	**	0.0023	7.355
NC:APP vs. 2BC:WT	*	0.0196	5.537	****	<0.0001	10.67
NC:APP vs. 2BC:APP	***	0.0003	9.148	****	<0.0001	16.22
NC:APP vs. IP:WT	*	0.0405	4.928	***	0.0003	9.246
NC:APP vs. IP:APP	***	0.001	8.133	****	<0.0001	11.38
2BC:WT vs. 2BC:APP	ns	0.1832	3.611	*	0.0194	5.546
2BC:WT vs. IP:WT	ns	0.9976	0.6094	ns	0.9066	1.425
2BC:WT vs. IP:APP	ns	0.481	2.595	ns	0.9952	0.709
2BC:APP vs. IP:WT	ns	0.0929	4.22	**	0.0036	6.971
2BC:APP vs. IP:APP	ns	0.9759	1.016	*	0.0451	4.837
IP:WT vs. IP:APP	ns	0.2784	3.205	ns	0.6655	2.134

FIGURE4

**FIGURE4**	**B**			**C**		
		P	t/F		P	t/F
n		3			3	
interaction	ns	F (2, 12) = 1.653	P=0.2322	ns	F (2, 12) = 1.682	P=0.2271
row factor	*	F (2, 12) = 4.994	P=0.0264	**	F (2, 12) = 12.14	P=0.0013
column factor	ns	F (1, 12) = 3.015	P=0.1081	*	F (1, 12) = 6.898	P=0.0221
method	two way ANOVA			two way ANOVA		
	Tukey					
NC:WT vs. NC:APP	ns	0.9998	0.3528	ns	>0.9999	0.2918
NC:WT vs. 2BC:WT	ns	0.9251	1.344	ns	0.8299	1.694
NC:WT vs. 2BC:APP	ns	0.058	4.624	*	0.017	5.653
NC:WT vs. IP:WT	ns	0.9935	0.7547	ns	0.1496	3.798
NC:WT vs. IP:APP	ns	0.6873	2.08	*	0.0115	5.98
NC:APP vs. 2BC:WT	ns	0.8291	1.696	ns	0.9121	1.402
NC:APP vs. 2BC:APP	*	0.0382	4.977	*	0.0241	5.361
NC:APP vs. IP:WT	ns	0.9653	1.107	ns	0.2047	3.506
NC:APP vs. IP:APP	ns	0.5447	2.433	*	0.0163	5.689
2BC:WT vs. 2BC:APP	ns	0.2581	3.281	ns	0.125	3.959
2BC:WT vs. IP:WT	ns	0.998	0.5889	ns	0.6778	2.104
2BC:WT vs. IP:APP	ns	0.9942	0.7364	ns	0.0861	4.287
2BC:APP vs. IP:WT	ns	0.1382	3.87	ns	0.7738	1.855
2BC:APP vs. IP:APP	ns	0.5006	2.544	ns	0.9999	0.3275
IP:WT vs. IP:APP	ns	0.9289	1.325	ns	0.646	2.183
FIGURE4	**F**			**I**		
		P	t/F		P	t/F
n		3			3	
interaction	*	F (2, 12) = 4.128	P=0.0432	ns	F (2, 12) = 3.802	P=0.0526
row factor	*	F (2, 12) = 4.543	P=0.0340	***	F (2, 12) = 16.05	P=0.0004
column factor	ns	F (1, 12) = 0.7324	P=0.4089	*	F (1, 12) = 7.088	P=0.0207
method	two way ANOVA			two way ANOVA		
NC:WT vs. NC:APP	ns	0.7243	1.986	ns	0.9981	0.5789
NC:WT vs. 2BC:WT	ns	0.8511	1.627	ns	0.996	0.6805
NC:WT vs. 2BC:APP	ns	0.7267	1.98	*	0.0178	5.617
NC:WT vs. IP:WT	ns	0.9992	0.4855	ns	0.09	4.248
NC:WT vs. IP:APP	ns	0.2677	3.244	**	0.0069	6.412
NC:APP vs. 2BC:WT	ns	0.1827	3.613	ns	0.9417	1.259
NC:APP vs. 2BC:APP	ns	0.124	3.966	**	0.0089	6.196
NC:APP vs. IP:WT	ns	0.8875	1.501	*	0.0456	4.827
NC:APP vs. IP:APP	*	0.0282	5.23	**	0.0035	6.991
2BC:WT vs. 2BC:APP	ns	0.9998	0.353	*	0.0401	4.936
2BC:WT vs. IP:WT	ns	0.6743	2.113	ns	0.1918	3.568
2BC:WT vs. IP:APP	ns	0.8542	1.617	*	0.0155	5.732
2BC:APP vs. IP:WT	ns	0.5316	2.466	ns	0.9197	1.369
2BC:APP vs. IP:APP	ns	0.9409	1.264	ns	0.9918	0.7955
IP:WT vs. IP:APP	ns	0.1612	3.729	ns	0.6535	2.164

FIGURE4	K			L		
		P	t/F		P	t/F
n		3			3	
interaction	ns	F (2, 12) = 0.6044	P=0.5622	*	F (2, 12) = 4.973	P=0.0267
row factor	***	F (2, 12) = 21.57	P=0.0001	****	F (2, 12) = 35.37	P<0.0001
column factor	**	F (1, 12) = 15.59	P=0.0019	ns	F (1, 12) = 0.02742	P=0.8712
method	two way ANOVA			two way ANOVA		
NC:WT vs. NC:APP	ns	0.673	2.116	ns	0.4825	2.591
NC:WT vs. 2BC:WT	ns	0.1205	3.992	**	0.0059	6.553
NC:WT vs. 2BC:APP	**	0.0027	7.232	*	0.0254	5.317
NC:WT vs. IP:WT	*	0.0267	5.276	ns	0.082	4.329
NC:WT vs. IP:APP	***	0.0002	9.59	**	0.0015	7.75
NC:APP vs. 2BC:WT	ns	0.766	1.876	***	0.0003	9.144
NC:APP vs. 2BC:APP	*	0.0323	5.117	**	0.0013	7.908
NC:APP vs. IP:WT	ns	0.2907	3.16	**	0.0038	6.92
NC:APP vs. IP:APP	**	0.002	7.475	***	0.0001	10.34
2BC:WT vs. 2BC:APP	ns	0.2687	3.24	ns	0.9458	1.236
2BC:WT vs. IP:WT	ns	0.9371	1.284	ns	0.629	2.224
2BC:WT vs. IP:APP	*	0.0182	5.598	ns	0.9523	1.197
2BC:APP vs. IP:WT	ns	0.7358	1.956	ns	0.9785	0.9884
2BC:APP vs. IP:APP	ns	0.5747	2.358	ns	0.5446	2.433
IP:WT vs. IP:APP	ns	0.0833	4.314	ns	0.2236	3.421

FIGURE4	N		
		P	t/F
n		3	
interaction	ns	F (2, 12) = 0.5882	P=0.5706
row factor	**	F (2, 12) = 9.998	P=0.0028
column factor	****	F (1, 12) = 34.95	P<0.0001
method	two way ANOVA		
NC:WT vs. NC:APP	*	0.0378	4.985
NC:WT vs. 2BC:WT	*	0.043	4.878
NC:WT vs. 2BC:APP	***	0.0006	8.55
NC:WT vs. IP:WT	ns	0.3485	2.969
NC:WT vs. IP:APP	***	0.0005	8.793
NC:APP vs. 2BC:WT	ns	>0.9999	0.107
NC:APP vs. 2BC:APP	ns	0.1925	3.565
NC:APP vs. IP:WT	ns	0.7126	2.016
NC:APP vs. IP:APP	ns	0.148	3.807
2BC:WT vs. 2BC:APP	ns	0.1716	3.672
2BC:WT vs. IP:WT	ns	0.7538	1.909
2BC:WT vs. IP:APP	ns	0.1314	3.914
2BC:APP vs. IP:WT	*	0.0186	5.581
2BC:APP vs. IP:APP	ns	>0.9999	0.2427
IP:WT vs. IP:APP	*	0.0139	5.823

FIGURE5						
**FIGURE5**	**C**			**D**		
n		5			5	
method		Multiple unpaired t tests			Multiple unpaired t tests	
	Correct for multiple comparisons using the Holm-sidak method					
		t ratio	Adjusted P Value		t ratio	Adjusted P Value
WT-APP	*	2.629	0.035421	ns	1.569	0.155292
APP/PS1-[illegible]	****	8.775	0.000089	****	9.056	0.000071
Ethanol-N	***	6.756	0.000432	***	6.5	0.000564
APP/PS1-[illegible]	*	2.97	0.035421	*	3.556	0.014824
**FIGURE5**	**E**			**F**		
n		6			6	
method		Multiple unpaired t tests			Multiple unpaired t tests	
		t ratio	Adjusted P Value		t ratio	Adjusted P Value
WT-APP	ns	0.3843	0.70882	ns	1.3	0.22281
APP/PS1-[illegible]	***	5.831	0.000663	****	9.045	0.000016
Ethanol-N	**	4.044	0.007024	**	5.314	0.001022
APP/PS1-[illegible]	*	2.804	0.036986	*	3.481	0.011797
**FIGURE5**	**G**					
n		6				
method		Multiple unpaired t tests				
		t ratio	Adjusted P Value			
WT-APP	ns	0.4829	0.639587			
APP/PS1-[illegible]	***	5.783	0.000708			
Ethanol-N	ns	2.301	0.086421			
APP/PS1-[illegible]	ns	2.677	0.068047			

FIGURE6						
**FIGURE6**	**C**			**D**		
n		5			5	
method		Multiple unpaired t tests			Multiple unpaired t tests	
	Correct for multiple comparisons using the Holm-sidak method					
		t ratio	Adjusted P Value		t ratio	Adjusted P Value
WT-APP	ns	0.07491	0.94388	ns	0.6817	0.532881
APP/PS1-[illegible]	**	10.6	0.001794	*	5.356	0.023247
Ethanol-N	ns	3.483	0.073951	*	4.265	0.038487
APP/PS1-[illegible]	ns	0.6316	0.808106	ns	1.434	0.399178

Supplementary figure1						
**Supplementary figure1**	**B**			**C**		
		P	t/F		P	t/F
n		3			3	
interaction	ns	F (4, 50) = 0.6368	P=0.6387	ns	F (4, 50) = 1.479	P=0.2225
row factor	ns	F (4, 50) = 1.234	P=0.3086	ns	F (4, 50) = 1.160	P=0.3398
column factor	ns	F (1, 50) = 1.641	P=0.2061	**	F (1, 50) = 9.568	P=0.0032
method	two way ANOVA			two way ANOVA		
	Tukey			Tukey		
NC:WT vs. NC:APP	ns	>0.9999	0.7915	ns	>0.9999	0.3397
NC:WT vs. DID:WT	ns	>0.9999	0.5181	ns	>0.9999	1.264
NC:WT vs. DID:APP	ns	>0.9999	0.1583	ns	>0.9999	0.5567
NC:WT vs. 2BC:WT	ns	>0.9999	0.9786	ns	>0.9999	0.3397
NC:WT vs. 2BC:APP	ns	0.9977	1.554	ns	0.9598	1.859
NC:WT vs. CIE:WT	ns	>0.9999	0.3016	ns	0.9047	2
NC:WT vs. CIE:APP	ns	>0.9999	0.9354	ns	>0.9999	0.6134
NC:WT vs. IP:WT	ns	>0.9999	0.6908	ns	>0.9999	0.4246
NC:WT vs. IP:APP	ns	0.7487	2.231	ns	>0.9999	0.1982
NC:APP vs. DID:WT	ns	>0.9999	0.2734	ns	>0.9999	0.9248
NC:APP vs. DID:APP	ns	>0.9999	0.9498	ns	>0.9999	0.8964
NC:APP vs. 2BC:WT	ns	>0.9999	0.1871	ns	>0.9999	0
NC:APP vs. 2BC:APP	ns	>0.9999	0.7627	ns	0.7745	2.199
NC:APP vs. CIE:WT	ns	>0.9999	0.4899	ns	0.9925	1.661
NC:APP vs. CIE:APP	ns	>0.9999	0.1439	ns	>0.9999	0.9531
NC:APP vs. IP:WT	ns	>0.9999	0.1007	ns	>0.9999	0.08493
NC:APP vs. IP:APP	ns	0.9995	1.439	ns	>0.9999	0.5379
DID:WT vs. DID:APP	ns	>0.9999	0.6764	ns	0.9694	1.821
DID:WT vs. 2BC:WT	ns	>0.9999	0.4605	ns	>0.9999	0.9248
DID:WT vs. 2BC:APP	ns	>0.9999	1.036	ns	0.1254	3.123
DID:WT vs. CIE:WT	ns	>0.9999	0.2164	ns	>0.9999	0.736
DID:WT vs. CIE:APP	ns	>0.9999	0.4173	ns	0.9542	1.878
DID:WT vs. IP:WT	ns	>0.9999	0.1727	ns	>0.9999	0.8398
DID:WT vs. IP:APP	ns	0.9876	1.713	ns	0.9993	1.463
DID:APP vs. 2BC:WT	ns	>0.9999	1.137	ns	>0.9999	0.8964
DID:APP vs. 2BC:APP	ns	0.9876	1.713	ns	>0.9999	1.302
DID:APP vs. CIE:WT	ns	>0.9999	0.4599	ns	0.4608	2.557
DID:APP vs. CIE:APP	ns	>0.9999	1.094	ns	>0.9999	0.05662
DID:APP vs. IP:WT	ns	>0.9999	0.8491	ns	>0.9999	0.9814
DID:APP vs. IP:APP	ns	0.6101	2.389	ns	>0.9999	0.3586
2BC:WT vs. 2BC:APP	ns	>0.9999	0.5756	ns	0.7745	2.199
2BC:WT vs. CIE:WT	ns	>0.9999	0.6769	ns	0.9925	1.661
2BC:WT vs. CIE:APP	ns	>0.9999	0.04317	ns	>0.9999	0.9531
2BC:WT vs. IP:WT	ns	>0.9999	0.2878	ns	>0.9999	0.08493
2BC:WT vs. IP:APP	ns	>0.9999	1.252	ns	>0.9999	0.5379
2BC:APP vs. CIE:WT	ns	>0.9999	1.253	*	0.0146	3.859
2BC:APP vs. CIE:APP	ns	>0.9999	0.6188	ns	>0.9999	1.246
2BC:APP vs. IP:WT	ns	>0.9999	0.8634	ns	0.7039	2.284
2BC:APP vs. IP:APP	ns	>0.9999	0.6764	ns	0.9925	1.661
CIE:WT vs. CIE:APP	ns	>0.9999	0.6338	ns	0.4138	2.614
CIE:WT vs. IP:WT	ns	>0.9999	0.3891	ns	0.997	1.576
CIE:WT vs. IP:APP	ns	0.9365	1.929	ns	0.7745	2.199
CIE:APP vs. IP:WT	ns	>0.9999	0.2446	ns	>0.9999	1.038
CIE:APP vs. IP:APP	ns	>0.9999	1.295	ns	>0.9999	0.4152
IP:WT vs. IP:APP	ns	0.9981	1.54	ns	>0.9999	0.6228

Supplementary figure1	D			E		
		P	t/F		P	t/F
n		3			3	
interaction	***	F (4, 50) = 5.631	P=0.0008	*	F (4, 50) = 2.612	P=0.0463
row factor	*	F (4, 50) = 3.078	P=0.0242	ns	F (4, 50) = 1.407	P=0.2453
column factor	**	F (1, 50) = 12.21	P=0.0010	**	F (1, 50) = 8.088	P=0.0064
method	two way ANOVA			two way ANOVA		
	Tukey			Tukey		
NC:WT vs. NC:APP	ns	0.9997	1.416	ns	0.3923	2.641
NC:WT vs. DID:WT	ns	>0.9999	0.7814	ns	>0.9999	0.03035
NC:WT vs. DID:APP	ns	0.9955	1.612	ns	0.0558	3.415
NC:WT vs. 2BC:WT	ns	>0.9999	1.123	ns	>0.9999	1.305
NC:WT vs. 2BC:APP	**	0.0017	4.526	ns	0.9999	1.366
NC:WT vs. CIE:WT	ns	>0.9999	0.3256	ns	>0.9999	0.5919
NC:WT vs. CIE:APP	ns	>0.9999	0.114	ns	>0.9999	0.5464
NC:WT vs. IP:WT	ns	>0.9999	0.1628	ns	>0.9999	0.1366
NC:WT vs. IP:APP	ns	>0.9999	0.4558	ns	>0.9999	0.4553
NC:APP vs. DID:WT	ns	>0.9999	0.6349	ns	0.4165	2.61
NC:APP vs. DID:APP	ns	>0.9999	0.1954	ns	>0.9999	0.774
NC:APP vs. 2BC:WT	ns	0.4759	2.54	ns	>0.9999	1.336
NC:APP vs. 2BC:APP	ns	0.1301	3.109	ns	>0.9999	1.275
NC:APP vs. CIE:WT	ns	0.9839	1.742	ns	0.8784	2.049
NC:APP vs. CIE:APP	ns	0.9983	1.53	ns	0.8502	2.094
NC:APP vs. IP:WT	ns	0.9969	1.579	ns	0.5067	2.504
NC:APP vs. IP:APP	ns	0.956	1.872	ns	0.7848	2.185
DID:WT vs. DID:APP	ns	>0.9999	0.8303	ns	0.0609	3.384
DID:WT vs. 2BC:WT	ns	0.9454	1.905	ns	>0.9999	1.275
DID:WT vs. 2BC:APP	*	0.0209	3.744	ns	>0.9999	1.336
DID:WT vs. CIE:WT	ns	>0.9999	1.107	ns	>0.9999	0.5615
DID:WT vs. CIE:APP	ns	>0.9999	0.8954	ns	>0.9999	0.516
DID:WT vs. IP:WT	ns	>0.9999	0.9442	ns	>0.9999	0.1062
DID:WT vs. IP:APP	ns	>0.9999	1.237	ns	>0.9999	0.425
DID:APP vs. 2BC:WT	ns	0.3223	2.735	ns	0.8401	2.11
DID:APP vs. 2BC:APP	ns	0.2135	2.914	ns	0.8784	2.049
DID:APP vs. CIE:WT	ns	0.9332	1.937	ns	0.2648	2.823
DID:APP vs. CIE:APP	ns	0.9861	1.726	ns	0.2382	2.868
DID:APP vs. IP:WT	ns	0.9788	1.774	ns	0.0822	3.278
DID:APP vs. IP:APP	ns	0.8673	2.067	ns	0.191	2.96
2BC:WT vs. 2BC:APP	****	<0.0001	5.649	ns	>0.9999	0.06071
2BC:WT vs. CIE:WT	ns	>0.9999	0.7977	ns	>0.9999	0.7133
2BC:WT vs. CIE:APP	ns	>0.9999	1.009	ns	>0.9999	0.7588
2BC:WT vs. IP:WT	ns	>0.9999	0.9605	ns	>0.9999	1.169
2BC:WT vs. IP:APP	ns	>0.9999	0.6675	ns	>0.9999	0.8499
2BC:APP vs. CIE:WT	***	0.0006	4.851	ns	>0.9999	0.774
2BC:APP vs. CIE:APP	**	0.0011	4.64	ns	>0.9999	0.8196
2BC:APP vs. IP:WT	***	0.001	4.688	ns	>0.9999	1.229
2BC:APP vs. IP:APP	***	0.0004	4.982	ns	>0.9999	0.9106
CIE:WT vs. CIE:APP	ns	>0.9999	0.2116	ns	>0.9999	0.04553
CIE:WT vs. IP:WT	ns	>0.9999	0.1628	ns	>0.9999	0.4553
CIE:WT vs. IP:APP	ns	>0.9999	0.1302	ns	>0.9999	0.1366
CIE:APP vs. IP:WT	ns	>0.9999	0.04884	ns	>0.9999	0.4098
CIE:APP vs. IP:APP	ns	>0.9999	0.3419	ns	>0.9999	0.09106
IP:WT vs. IP:APP	ns	>0.9999	0.293	ns	>0.9999	0.3187

Supplementary figure1	F			H		
		P	t/F		P	t/F
n		3			3	
interaction	ns	F (4, 50) = 0.3772	P=0.8238	*	F (2, 12) = 4.973	P=0.0267
row factor	ns	F (4, 50) = 0.1143	P=0.9769	****	F (2, 12) = 35.37	P<0.0001
column factor	ns	F (1, 50) = 0.07239	P=0.7890	ns	F (1, 12) = 0.02742	P=0.8712
method	two way ANOVA			two way ANOVA		
	Tukey			Tukey		
NC:WT vs. NC:APP	ns	>0.9999	0.1119	ns	>0.9999	0.3387
NC:WT vs. DID:WT	ns	>0.9999	0.3358	ns	>0.9999	0.5757
NC:WT vs. DID:APP	ns	>0.9999	0.1119	ns	>0.9999	0.9821
NC:WT vs. 2BC:WT	ns	>0.9999	0.1119	ns	>0.9999	0.09031
NC:WT vs. 2BC:APP	ns	>0.9999	0.8255	ns	>0.9999	0.8579
NC:WT vs. CIE:WT	ns	>0.9999	0.1679	ns	>0.9999	0.01129
NC:WT vs. CIE:APP	ns	>0.9999	0.05596	ns	>0.9999	0.4854
NC:WT vs. IP:WT	ns	>0.9999	0.6996	ns	>0.9999	0.198
NC:WT vs. IP:APP	ns	>0.9999	0.2798	ns	0.9406	1.919
NC:APP vs. DID:WT	ns	>0.9999	0.2239	ns	>0.9999	0.2371
NC:APP vs. DID:APP	ns	>0.9999	0	ns	>0.9999	1.321
NC:APP vs. 2BC:WT	ns	>0.9999	0	ns	>0.9999	0.2483
NC:APP vs. 2BC:APP	ns	>0.9999	0.7135	ns	>0.9999	0.5193
NC:APP vs. CIE:WT	ns	>0.9999	0.05596	ns	>0.9999	0.3274
NC:APP vs. CIE:APP	ns	>0.9999	0.1679	ns	>0.9999	0.824
NC:APP vs. IP:WT	ns	>0.9999	0.5876	ns	>0.9999	0.1249
NC:APP vs. IP:APP	ns	>0.9999	0.3918	ns	0.9969	1.58
DID:WT vs. DID:APP	ns	>0.9999	0.2239	ns	0.9976	1.558
DID:WT vs. 2BC:WT	ns	>0.9999	0.2239	ns	>0.9999	0.4854
DID:WT vs. 2BC:APP	ns	>0.9999	0.4897	ns	>0.9999	0.2822
DID:WT vs. CIE:WT	ns	>0.9999	0.1679	ns	>0.9999	0.5644
DID:WT vs. CIE:APP	ns	>0.9999	0.3918	ns	>0.9999	1.061
DID:WT vs. IP:WT	ns	>0.9999	0.3638	ns	>0.9999	0.3509
DID:WT vs. IP:APP	ns	>0.9999	0.6156	ns	>0.9999	1.343
DID:APP vs. 2BC:WT	ns	>0.9999	0	ns	>0.9999	1.072
DID:APP vs. 2BC:APP	ns	>0.9999	0.7135	ns	0.9651	1.84
DID:APP vs. CIE:WT	ns	>0.9999	0.05596	ns	>0.9999	0.9934
DID:APP vs. CIE:APP	ns	>0.9999	0.1679	ns	>0.9999	0.4967
DID:APP vs. IP:WT	ns	>0.9999	0.5876	ns	>0.9999	1.134
DID:APP vs. IP:APP	ns	>0.9999	0.3918	ns	0.2217	2.901
2BC:WT vs. 2BC:APP	ns	>0.9999	0.7135	ns	>0.9999	0.7676
2BC:WT vs. CIE:WT	ns	>0.9999	0.05596	ns	>0.9999	0.07902
2BC:WT vs. CIE:APP	ns	>0.9999	0.1679	ns	>0.9999	0.5757
2BC:WT vs. IP:WT	ns	>0.9999	0.5876	ns	>0.9999	0.1119
2BC:WT vs. IP:APP	ns	>0.9999	0.3918	ns	0.9678	1.829
2BC:APP vs. CIE:WT	ns	>0.9999	0.6576	ns	>0.9999	0.8466
2BC:APP vs. CIE:APP	ns	>0.9999	0.8814	ns	>0.9999	1.343
2BC:APP vs. IP:WT	ns	>0.9999	0.1259	ns	>0.9999	0.6199
2BC:APP vs. IP:APP	ns	>0.9999	1.105	ns	>0.9999	1.061
CIE:WT vs. CIE:APP	ns	>0.9999	0.2239	ns	>0.9999	0.4967
CIE:WT vs. IP:WT	ns	>0.9999	0.5317	ns	>0.9999	0.1873
CIE:WT vs. IP:APP	ns	>0.9999	0.4477	ns	0.9447	1.908
CIE:APP vs. IP:WT	ns	>0.9999	0.7555	ns	>0.9999	0.6608
CIE:APP vs. IP:APP	ns	>0.9999	0.2239	ns	0.5975	2.404
IP:WT vs. IP:APP	ns	>0.9999	0.9794	ns	0.9945	1.632

Supplementary figure1	I			J		
		P	t/F		P	t/F
n		3			3	
interaction	ns	F (4, 49) = 0.3412	P=0.8488	ns	F (4, 49) = 0.2814	P=0.8887
row factor	**	F (4, 49) = 4.949	P=0.0020	***	F (4, 49) = 6.103	P=0.0005
column factor	ns	F (1, 49) = 0.1944	P=0.6613	*	F (1, 49) = 4.453	P=0.0400
method	two way ANOVA			two way ANOVA		
	Tukey			Tukey		
NC:WT vs. NC:APP	ns	>0.9999	0.3195	ns	>0.9999	1.288
NC:WT vs. DID:WT	ns	>0.9999	0.4741	ns	>0.9999	1.107
NC:WT vs. DID:APP	ns	>0.9999	0.1134	ns	>0.9999	1.254
NC:WT vs. 2BC:WT	ns	0.859	2.082	ns	0.5348	2.474
NC:WT vs. 2BC:APP	ns	0.1698	3.009	**	0.0092	4.011
NC:WT vs. CIE:WT	ns	>0.9999	1.196	ns	>0.9999	0.6779
NC:WT vs. CIE:APP	ns	>0.9999	0.7627	ns	0.9917	1.672
NC:WT vs. IP:WT	ns	0.9155	1.979	ns	0.3292	2.727
NC:WT vs. IP:APP	ns	0.4122	2.618	*	0.0273	3.66
NC:APP vs. DID:WT	ns	>0.9999	0.1546	ns	>0.9999	0.1808
NC:APP vs. DID:APP	ns	>0.9999	0.2061	ns	>0.9999	0.03389
NC:APP vs. 2BC:WT	ns	0.9809	1.762	ns	>0.9999	1.186
NC:APP vs. 2BC:APP	ns	0.3564	2.69	ns	0.3325	2.723
NC:APP vs. CIE:WT	ns	>0.9999	0.876	ns	>0.9999	0.6101
NC:APP vs. CIE:APP	ns	>0.9999	0.4432	ns	>0.9999	0.3841
NC:APP vs. IP:WT	ns	0.9914	1.674	ns	0.9989	1.499
NC:APP vs. IP:APP	ns	0.6923	2.298	ns	0.6263	2.373
DID:WT vs. DID:APP	ns	>0.9999	0.3607	ns	>0.9999	0.1469
DID:WT vs. 2BC:WT	ns	0.9958	1.608	ns	0.9999	1.367
DID:WT vs. 2BC:APP	ns	0.4811	2.535	ns	0.2205	2.904
DID:WT vs. CIE:WT	ns	>0.9999	0.7214	ns	>0.9999	0.4293
DID:WT vs. CIE:APP	ns	>0.9999	0.2886	ns	>0.9999	0.5649
DID:WT vs. IP:WT	ns	0.9984	1.527	ns	0.9917	1.672
DID:WT vs. IP:APP	ns	0.8171	2.144	ns	0.4658	2.553
DID:APP vs. 2BC:WT	ns	0.9204	1.969	ns	>0.9999	1.22
DID:APP vs. 2BC:APP	ns	0.2244	2.896	ns	0.309	2.757
DID:APP vs. CIE:WT	ns	>0.9999	1.082	ns	>0.9999	0.5762
DID:APP vs. CIE:APP	ns	>0.9999	0.6493	ns	>0.9999	0.418
DID:APP vs. IP:WT	ns	0.9566	1.871	ns	0.9983	1.532
DID:APP vs. IP:APP	ns	0.5081	2.504	ns	0.5957	2.406
2BC:WT vs. 2BC:APP	ns	>0.9999	0.9276	ns	0.9982	1.536
2BC:WT vs. CIE:WT	ns	>0.9999	0.8863	ns	0.9749	1.796
2BC:WT vs. CIE:APP	ns	>0.9999	1.319	ns	>0.9999	0.8021
2BC:WT vs. IP:WT	ns	>0.9999	0.005896	ns	>0.9999	0.3684
2BC:WT vs. IP:APP	ns	>0.9999	0.5359	ns	>0.9999	1.186
2BC:APP vs. CIE:WT	ns	0.9712	1.814	ns	0.0713	3.333
2BC:APP vs. CIE:APP	ns	0.7364	2.247	ns	0.6567	2.339
2BC:APP vs. IP:WT	ns	>0.9999	0.8903	ns	>0.9999	1.097
2BC:APP vs. IP:APP	ns	>0.9999	0.3916	ns	>0.9999	0.3502
CIE:WT vs. CIE:APP	ns	>0.9999	0.4329	ns	>0.9999	0.9942
CIE:WT vs. IP:WT	ns	>0.9999	0.8392	ns	0.8595	2.081
CIE:WT vs. IP:APP	ns	0.9996	1.422	ns	0.1816	2.983
CIE:APP vs. IP:WT	ns	>0.9999	1.252	ns	>0.9999	1.133
CIE:APP vs. IP:APP	ns	0.9611	1.855	ns	0.9111	1.988
IP:WT vs. IP:APP	ns	>0.9999	0.5169	ns	>0.9999	0.7627

Supplementary figure1	K			L		
		P	t/F		P	t/F
n		3			3	
interaction	ns	F (4, 49) = 1.084	P=0.3747	ns	F (4, 49) = 1.338	P=0.2692
row factor	**	F (4, 49) = 4.802	P=0.0024	**	F (4, 49) = 4.723	P=0.0026
column factor	ns	F (1, 49) = 2.777	P=0.1020	ns	F (1, 49) = 2.977	P=0.0908
method	two way ANOVA			two way ANOVA		
	Tukey			Tukey		
NC:WT vs. NC:APP	ns	>0.9999	0.3283	ns	>0.9999	0.5151
NC:WT vs. DID:WT	ns	>0.9999	0.8206	ns	>0.9999	0.1405
NC:WT vs. DID:APP	ns	>0.9999	0.6275	ns	>0.9999	0.04683
NC:WT vs. 2BC:WT	ns	>0.9999	1.313	ns	0.9999	1.358
NC:WT vs. 2BC:APP	ns	0.0963	3.225	ns	0.2204	2.904
NC:WT vs. CIE:WT	ns	>0.9999	0.4827	ns	>0.9999	0.7025
NC:WT vs. CIE:APP	ns	>0.9999	1.101	ns	>0.9999	0.2342
NC:WT vs. IP:WT	ns	0.999	1.493	ns	>0.9999	1.152
NC:WT vs. IP:APP	ns	0.0737	3.321	*	0.0322	3.606
NC:APP vs. DID:WT	ns	>0.9999	1.149	ns	>0.9999	0.3747
NC:APP vs. DID:APP	ns	>0.9999	0.9558	ns	>0.9999	0.4683
NC:APP vs. 2BC:WT	ns	0.9939	1.641	ns	>0.9999	0.843
NC:APP vs. 2BC:APP	*	0.0377	3.553	ns	0.612	2.388
NC:APP vs. CIE:WT	ns	>0.9999	0.811	ns	>0.9999	0.1873
NC:APP vs. CIE:APP	ns	0.9996	1.429	ns	>0.9999	0.281
NC:APP vs. IP:WT	ns	0.9729	1.806	ns	>0.9999	0.6609
NC:APP vs. IP:APP	*	0.0283	3.649	ns	0.1377	3.091
DID:WT vs. DID:APP	ns	>0.9999	0.1931	ns	>0.9999	0.09366
DID:WT vs. 2BC:WT	ns	>0.9999	0.4924	ns	>0.9999	1.218
DID:WT vs. 2BC:APP	ns	0.5979	2.404	ns	0.3047	2.763
DID:WT vs. CIE:WT	ns	>0.9999	0.3379	ns	>0.9999	0.562
DID:WT vs. CIE:APP	ns	>0.9999	0.28	ns	>0.9999	0.09366
DID:WT vs. IP:WT	ns	>0.9999	0.7106	ns	>0.9999	1.018
DID:WT vs. IP:APP	ns	0.5115	2.501	*	0.0487	3.466
DID:APP vs. 2BC:WT	ns	>0.9999	0.6855	ns	>0.9999	1.311
DID:APP vs. 2BC:APP	ns	0.4291	2.597	ns	0.2463	2.857
DID:APP vs. CIE:WT	ns	>0.9999	0.1448	ns	>0.9999	0.6556
DID:APP vs. CIE:APP	ns	>0.9999	0.4731	ns	>0.9999	0.1873
DID:APP vs. IP:WT	ns	>0.9999	0.8947	ns	>0.9999	1.107
DID:APP vs. IP:APP	ns	0.3536	2.694	*	0.037	3.559
2BC:WT vs. 2BC:APP	ns	0.9433	1.912	ns	0.998	1.545
2BC:WT vs. CIE:WT	ns	>0.9999	0.8303	ns	>0.9999	0.6556
2BC:WT vs. CIE:APP	ns	>0.9999	0.2124	ns	>0.9999	1.124
2BC:WT vs. IP:WT	ns	>0.9999	0.2412	ns	>0.9999	0.1429
2BC:WT vs. IP:APP	ns	0.9013	2.008	ns	0.7354	2.248
2BC:APP vs. CIE:WT	ns	0.3191	2.742	ns	0.7736	2.201
2BC:APP vs. CIE:APP	ns	0.831	2.124	ns	0.3718	2.669
2BC:APP vs. IP:WT	ns	0.9969	1.581	ns	0.9953	1.616
2BC:APP vs. IP:APP	ns	>0.9999	0.09655	ns	>0.9999	0.7025
CIE:WT vs. CIE:APP	ns	>0.9999	0.6179	ns	>0.9999	0.4683
CIE:WT vs. IP:WT	ns	>0.9999	1.033	ns	>0.9999	0.4822
CIE:WT vs. IP:APP	ns	0.257	2.838	ns	0.2204	2.904
CIE:APP vs. IP:WT	ns	>0.9999	0.4437	ns	>0.9999	0.9288
CIE:APP vs. IP:APP	ns	0.758	2.221	ns	0.0638	3.372
IP:WT vs. IP:APP	ns	0.9915	1.674	ns	0.7028	2.286

Supplementary figure1	H			I		
		P	t/F		P	t/F
n		3			3	
interaction	ns	F (4, 49) = 1.338	P=0.2692	ns	F (4, 50) = 0.2432	P=0.9124
row factor	**	F (4, 49) = 4.723	P=0.0026	****	F (4, 50) = 7.956	P<0.0001
column factor	ns	F (1, 49) = 2.977	P=0.0908	ns	F (1, 50) = 1.457	P=0.2331
method	two way ANOVA			two way ANOVA		
	Tukey			Tukey		
NC:WT vs. NC:APP	ns	>0.9999	0.5151	ns	>0.9999	0.7762
NC:WT vs. DID:WT	ns	>0.9999	0.1405	ns	>0.9999	0.8956
NC:WT vs. DID:APP	ns	>0.9999	0.04683	ns	>0.9999	1.278
NC:WT vs. 2BC:WT	ns	0.9999	1.358	ns	0.0986	3.212
NC:WT vs. 2BC:APP	ns	0.2204	2.904	**	0.0087	4.024
NC:WT vs. CIE:WT	ns	>0.9999	0.7025	ns	0.9433	1.911
NC:WT vs. CIE:APP	ns	>0.9999	0.2342	ns	0.9926	1.66
NC:WT vs. IP:WT	ns	>0.9999	1.152	ns	0.2328	2.878
NC:WT vs. IP:APP	*	0.0322	3.606	*	0.0147	3.857
NC:APP vs. DID:WT	ns	>0.9999	0.3747	ns	>0.9999	0.1194
NC:APP vs. DID:APP	ns	>0.9999	0.4683	ns	>0.9999	0.5016
NC:APP vs. 2BC:WT	ns	>0.9999	0.843	ns	0.5674	2.436
NC:APP vs. 2BC:APP	ns	0.612	2.388	ns	0.0894	3.248
NC:APP vs. CIE:WT	ns	>0.9999	0.1873	ns	>0.9999	1.134
NC:APP vs. CIE:APP	ns	>0.9999	0.281	ns	>0.9999	0.8837
NC:APP vs. IP:WT	ns	>0.9999	0.6609	ns	0.8454	2.102
NC:APP vs. IP:APP	ns	0.1377	3.091	ns	0.1402	3.081
DID:WT vs. DID:APP	ns	>0.9999	0.09366	ns	>0.9999	0.3821
DID:WT vs. 2BC:WT	ns	>0.9999	1.218	ns	0.6748	2.317
DID:WT vs. 2BC:APP	ns	0.3047	2.763	ns	0.1236	3.129
DID:WT vs. CIE:WT	ns	>0.9999	0.562	ns	>0.9999	1.015
DID:WT vs. CIE:APP	ns	>0.9999	0.09366	ns	>0.9999	0.7643
DID:WT vs. IP:WT	ns	>0.9999	1.018	ns	0.9135	1.982
DID:WT vs. IP:APP	*	0.0487	3.466	ns	0.19	2.962
DID:APP vs. 2BC:WT	ns	>0.9999	1.311	ns	0.9343	1.935
DID:APP vs. 2BC:APP	ns	0.2463	2.857	ns	0.3143	2.747
DID:APP vs. CIE:WT	ns	>0.9999	0.6556	ns	>0.9999	0.6329
DID:APP vs. CIE:APP	ns	>0.9999	0.1873	ns	>0.9999	0.3821
DID:APP vs. IP:WT	ns	>0.9999	1.107	ns	0.9961	1.6
DID:APP vs. IP:APP	*	0.037	3.559	ns	0.4421	2.579
2BC:WT vs. 2BC:APP	ns	0.998	1.545	ns	>0.9999	0.812
2BC:WT vs. CIE:WT	ns	>0.9999	0.6556	ns	>0.9999	1.302
2BC:WT vs. CIE:APP	ns	>0.9999	1.124	ns	0.9978	1.552
2BC:WT vs. IP:WT	ns	>0.9999	0.1429	ns	>0.9999	0.3344
2BC:WT vs. IP:APP	ns	0.7354	2.248	ns	>0.9999	0.6449
2BC:APP vs. CIE:WT	ns	0.7736	2.201	ns	0.8373	2.114
2BC:APP vs. CIE:APP	ns	0.3718	2.669	ns	0.6321	2.364
2BC:APP vs. IP:WT	ns	0.9953	1.616	ns	>0.9999	1.146
2BC:APP vs. IP:APP	ns	>0.9999	0.7025	ns	>0.9999	0.1672
CIE:WT vs. CIE:APP	ns	>0.9999	0.4683	ns	>0.9999	0.2508
CIE:WT vs. IP:WT	ns	>0.9999	0.4822	ns	>0.9999	0.9673
CIE:WT vs. IP:APP	ns	0.2204	2.904	ns	0.9295	1.947
CIE:APP vs. IP:WT	ns	>0.9999	0.9288	ns	>0.9999	1.218
CIE:APP vs. IP:APP	ns	0.0638	3.372	ns	0.7756	2.197
IP:WT vs. IP:APP	ns	0.7028	2.286	ns	>0.9999	0.9792

Supplementary figure1	J			K		
		P	t/F		P	t/F
n		3			3	
interaction	ns	F (4, 50) = 1.175	P=0.3332	ns	F (4, 50) = 0.2146	P=0.9291
row factor	****	F (4, 50) = 32.23	P<0.0001	****	F (4, 50) = 29.17	P<0.0001
column factor	**	F (1, 50) = 8.124	P=0.0063	ns	F (1, 50) = 1.793	P=0.1866
method	two way ANOVA			two way ANOVA		
	Tukey			Tukey		
NC:WT vs. NC:APP	ns	0.9978	1.553	ns	>0.9999	0.2629
NC:WT vs. DID:WT	ns	>0.9999	1.165	ns	>0.9999	0.04382
NC:WT vs. DID:APP	ns	>0.9999	0.7478	ns	>0.9999	0.1022
NC:WT vs. 2BC:WT	***	0.0004	4.947	***	0.0008	4.761
NC:WT vs. 2BC:APP	****	<0.0001	7.121	****	<0.0001	5.638
NC:WT vs. CIE:WT	ns	0.9609	1.855	ns	>0.9999	0.4966
NC:WT vs. CIE:APP	ns	0.3143	2.747	ns	>0.9999	0.08763
NC:WT vs. IP:WT	****	<0.0001	5.666	**	0.0089	4.017
NC:WT vs. IP:APP	****	<0.0001	7.837	***	0.0002	5.229
NC:APP vs. DID:WT	ns	>0.9999	0.3883	ns	>0.9999	0.2191
NC:APP vs. DID:APP	ns	>0.9999	0.8053	ns	>0.9999	0.1607
NC:APP vs. 2BC:WT	ns	0.0593	3.394	**	0.0018	4.498
NC:APP vs. 2BC:APP	****	<0.0001	5.568	****	<0.0001	5.375
NC:APP vs. CIE:WT	ns	>0.9999	0.302	ns	>0.9999	0.7595
NC:APP vs. CIE:APP	ns	>0.9999	1.194	ns	>0.9999	0.1753
NC:APP vs. IP:WT	**	0.0065	4.113	*	0.0203	3.754
NC:APP vs. IP:APP	****	<0.0001	6.284	***	0.0004	4.966
DID:WT vs. DID:APP	ns	>0.9999	0.417	ns	>0.9999	0.05842
DID:WT vs. 2BC:WT	*	0.0186	3.782	***	0.0009	4.718
DID:WT vs. 2BC:APP	****	<0.0001	5.956	****	<0.0001	5.594
DID:WT vs. CIE:WT	ns	>0.9999	0.6902	ns	>0.9999	0.5404
DID:WT vs. CIE:APP	ns	0.9968	1.582	ns	>0.9999	0.04382
DID:WT vs. IP:WT	**	0.0018	4.501	*	0.0102	3.973
DID:WT vs. IP:APP	****	<0.0001	6.672	***	0.0002	5.185
DID:APP vs. 2BC:WT	**	0.0049	4.199	**	0.0011	4.659
DID:APP vs. 2BC:APP	****	<0.0001	6.373	****	<0.0001	5.535
DID:APP vs. CIE:WT	ns	>0.9999	1.107	ns	>0.9999	0.5988
DID:APP vs. CIE:APP	ns	0.9055	1.999	ns	>0.9999	0.01461
DID:APP vs. IP:WT	***	0.0004	4.918	*	0.0123	3.914
DID:APP vs. IP:APP	****	<0.0001	7.089	***	0.0002	5.127
2BC:WT vs. 2BC:APP	ns	0.7935	2.174	ns	>0.9999	0.8763
2BC:WT vs. CIE:WT	ns	0.1363	3.092	***	0.0001	5.258
2BC:WT vs. CIE:APP	ns	0.7733	2.2	**	0.001	4.674
2BC:WT vs. IP:WT	ns	>0.9999	0.719	ns	>0.9999	0.7449
2BC:WT vs. IP:APP	ns	0.2261	2.89	ns	>0.9999	0.4674
2BC:APP vs. CIE:WT	***	0.0001	5.266	****	<0.0001	6.134
2BC:APP vs. CIE:APP	**	0.0028	4.374	****	<0.0001	5.55
2BC:APP vs. IP:WT	ns	0.9994	1.455	ns	0.995	1.621
2BC:APP vs. IP:APP	ns	>0.9999	0.7161	ns	>0.9999	0.409
CIE:WT vs. CIE:APP	ns	>0.9999	0.8916	ns	>0.9999	0.5842
CIE:WT vs. IP:WT	*	0.017	3.811	**	0.0018	4.513
CIE:WT vs. IP:APP	****	<0.0001	5.982	****	<0.0001	5.725
CIE:APP vs. IP:WT	ns	0.2109	2.919	*	0.0117	3.929
CIE:APP vs. IP:APP	***	0.0002	5.091	***	0.0002	5.141
IP:WT vs. IP:APP	ns	0.7957	2.171	ns	>0.9999	1.212

Supplementary figure1	L		
		P	t/F
n		3	
interaction	ns	F (4, 50) = 0.6019	P=0.6630
row factor	****	F (4, 50) = 17.13	P<0.0001
column factor	ns	F (1, 50) = 0.2632	P=0.6102
method	two way ANOVA		
	Tukey		
NC:WT vs. NC:APP	ns	>0.9999	0.0956
NC:WT vs. DID:WT	ns	>0.9999	0.8923
NC:WT vs. DID:APP	ns	>0.9999	0.2231
NC:WT vs. 2BC:WT	**	0.0081	4.047
NC:WT vs. 2BC:APP	***	0.0004	4.939
NC:WT vs. CIE:WT	ns	>0.9999	1.115
NC:WT vs. CIE:APP	ns	>0.9999	0.7967
NC:WT vs. IP:WT	ns	0.0566	3.41
NC:WT vs. IP:APP	**	0.0015	4.557
NC:APP vs. DID:WT	ns	>0.9999	0.7967
NC:APP vs. DID:APP	ns	>0.9999	0.1275
NC:APP vs. 2BC:WT	*	0.0109	3.952
NC:APP vs. 2BC:APP	***	0.0006	4.844
NC:APP vs. CIE:WT	ns	>0.9999	1.02
NC:APP vs. CIE:APP	ns	>0.9999	0.7011
NC:APP vs. IP:WT	ns	0.0743	3.314
NC:APP vs. IP:APP	**	0.0021	4.461
DID:WT vs. DID:APP	ns	>0.9999	0.6692
DID:WT vs. 2BC:WT	ns	0.1153	3.155
DID:WT vs. 2BC:APP	**	0.0081	4.047
DID:WT vs. CIE:WT	ns	>0.9999	0.2231
DID:WT vs. CIE:APP	ns	>0.9999	0.0956
DID:WT vs. IP:WT	ns	0.495	2.518
DID:WT vs. IP:APP	*	0.0266	3.665
DID:APP vs. 2BC:WT	*	0.0163	3.824
DID:APP vs. 2BC:APP	***	0.0009	4.716
DID:APP vs. CIE:WT	ns	>0.9999	0.8923
DID:APP vs. CIE:APP	ns	>0.9999	0.5736
DID:APP vs. IP:WT	ns	0.1058	3.187
DID:APP vs. IP:APP	**	0.0032	4.334
2BC:WT vs. 2BC:APP	ns	>0.9999	0.8923
2BC:WT vs. CIE:WT	ns	0.2045	2.932
2BC:WT vs. CIE:APP	ns	0.0888	3.25
2BC:WT vs. IP:WT	ns	>0.9999	0.6373
2BC:WT vs. IP:APP	ns	>0.9999	0.5099
2BC:APP vs. CIE:WT	*	0.0163	3.824
2BC:APP vs. CIE:APP	**	0.0059	4.143
2BC:APP vs. IP:WT	ns	0.9983	1.53
2BC:APP vs. IP:APP	ns	>0.9999	0.3824
CIE:WT vs. CIE:APP	ns	>0.9999	0.3187
CIE:WT vs. IP:WT	ns	0.6944	2.294
CIE:WT vs. IP:APP	ns	0.0516	3.442
CIE:APP vs. IP:WT	ns	0.4144	2.613
CIE:APP vs. IP:APP	*	0.0199	3.76
IP:WT vs. IP:APP	ns	>0.9999	1.147

Supplementary figure2

Row 1		Adjusted P Value	q	DF
APP/PS1 vs. WT	ns	0.5228	1.188	9.764
APP/PS1 vs. APP/PS1-Ethanol	ns	0.4148	1.383	9.152
APP/PS1 vs. APP/PS1-Ethanol+NAC	ns	0.2979	1.644	8.335
Row 2				
APP/PS1 vs. WT	ns	0.9961	0.1731	8.693
APP/PS1 vs. APP/PS1-Ethanol	ns	0.0739	2.611	8.107
APP/PS1 vs. APP/PS1-Ethanol+NAC	ns	0.5791	1.098	9.51
Row 3				
APP/PS1 vs. WT	ns	0.6803	0.9395	8.996
APP/PS1 vs. APP/PS1-Ethanol	**	0.0046	4.447	8.678
APP/PS1 vs. APP/PS1-Ethanol+NAC	ns	0.0652	2.646	8.932
Row 4				
APP/PS1 vs. WT	ns	0.9023	0.549	7.913
APP/PS1 vs. APP/PS1-Ethanol	**	0.008	4.001	9.03
APP/PS1 vs. APP/PS1-Ethanol+NAC	ns	0.0991	2.347	9.829
Row 5				
APP/PS1 vs. WT	ns	0.9972	0.155	9.987
APP/PS1 vs. APP/PS1-Ethanol	*	0.0191	3.355	9.843
APP/PS1 vs. APP/PS1-Ethanol+NAC	ns	0.443	1.327	9.667
